# A Bis-Cyclopentadienyl Ligand-Supported Di-Iron Trihydride Motif as a Synthon for Access to Heterobimetallic Trinuclear Complexes

**DOI:** 10.1021/acs.inorgchem.4c01420

**Published:** 2024-05-30

**Authors:** Chung-Ching Tseng, Yi-Wun Ding, Zhong-Yue Chen, Hao-Yuan Lan, Han-Jung Li, You-Song Cheng, Ting-Shen Kuo, Pei-Lin Chen, Wen-Chun Wu, Fong-Ku Shi, Tzuhsiung Yang, Hsueh-Ju Liu

**Affiliations:** † Department of Applied Chemistry, National Yang Ming Chiao Tung University, 1001 Daxue Rd, East District, Hsinchu City 300093, Taiwan; ‡ Center for Emergent Functional Matter Science, National Yang Ming Chiao Tung University, 1001 Daxue Rd, East District, Hsinchu City 300093, Taiwan; § Department of Chemistry, National Taiwan Normal University, Taipei 11677, Taiwan; ∥ Department of Chemistry, National Tsing Hua University, Hsinchu City 300044, Taiwan; ⊥ Rezwave Technology Inc., 3F-5, 79, Hsin Tai Wu Rd., Sec.1, HsiChih District, New Taipei City 221432, Taiwan

## Abstract

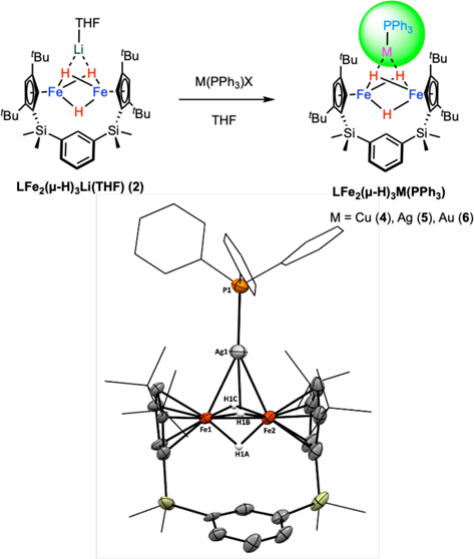

Herein, we report the synthesis of a flexible bis-cyclopentadienyl ligand **L** (the doubly deprotonated form of **H**_**2**_**L** (1,3-bis(2,4-di-*tert*-butylcyclopentadienyldimethylsilyl)benzene)), demonstrating its ability to stabilize a series of di-iron hydrido complexes. Notably, this ligand facilitates the isolation of an unprecedented anionic cyclopentadienyl ligand-supported di-iron trihydride complex, **LFe**_**2**_**(μ-H)**_**3**_**Li(THF)** (**2**), functioning as a synthon for the [Fe_2_(μ-H)_3_]^−^ core and providing access to heterobimetallic complexes **4**–**6** with coinage metals.

## Abstract 2

A newly synthesized bis-cyclopentadienyl ligand, denoted as L, facilitates the isolation of a novel anionic cyclopentadienyl ligand-supported di-iron trihydride complex, LFe_2_(μ-H)_3_Li(THF) (**2**). This complex serves as a synthon for the [Fe_2_(μ-H)_3_]^−^ core and serves as a precursor for the synthesis of a series of heterobimetallic complexes (**4**−**6**) featuring coinage metals.

## Introduction

Iron hydrides have been recognized as highly reactive intermediates in synthetic, biological, and industrial catalytic transformations.^1−9^ To gain a deeper understanding and model these reactive entities, molecular iron hydrides have been synthesized as focal points for research. These hydrides are stabilized by a range of ligand frameworks, including carbon monoxide,^10,11^ β-diketiminates,^12,13^ phosphines,^14^ NHC carbenes,^15,16^ pincer-type ligands,^3,4,9,17−19^ and other types of ligands. During synthesis, monomeric iron hydrido complexes with these ligands often lead to the formation of clusters, with hydride-bridged dimers being a common occurrence. Researchers have employed cyclopentadienyl derivatives (Cp^R^), a well-known class of ligands, to support iron hydride complexes. In the literature, iron hydride complexes supported exclusively by Cp^R^ ligands typically manifest as binuclear complexes featuring hydride bridges of the type [(Cp^R^Fe)_2_(μ-H)*_n_*], where ‘*n*’ is typically 3 or 4. Examples of such complexes include (Cp′Fe)_2_(μ-H)*_n_* (*n* = 3, **A**; *n* = 4, **B**; Cp′ = 1,2,4-C_5_H_2_^*t*^Bu_3_),^20^ (Cp*Fe)_2_(μ-H)_4_ (**C**; Cp* = C_5_Me_5_),^21^ (^5^CpFe)_2_(μ-H)_4_ (**D**; ^5^Cp = C_5_^*i*^Pr_5_),^22^ and (^4^CpFe)_2_(μ-H)_4_ (**E**; ^4^Cp = C_5_H^*i*^Pr_4_).^23^

Driven by our interest in metal hydride chemistry and multimetallic structures, we have previously employed pyrroles—ligands that are isoelectronic and nitrogen analogues of Cp^R^—to stabilize low-valent main group Sn^24^ and Pb^25^ hydrides and multimetallic frameworks containing metal–metal bonds.^26,27^ Given the extensive applications of this specific set of donors in organometallic and inorganic chemistry, and considering the frequent occurrence of multiple metal clusters in their reactions, our objective was to develop compounds in which an iron hydride cluster is supported by two prearranged Cp donors with sufficient steric protection to maintain low coordination numbers on iron centers. Tethering two Cp^R^ groups together results in a bis-Cp ligands that are suitable for accommodating two metal centers, forming two half-sandwiched metal complexes. By employing bis-Cp ligands, the proximity of two CpM fragments might encourage collaboration between metal centers, thus furnishing new structures, properties, and reactivity compared to the unlinked cyclopentadienyl complexes. While several bis-Cp ligands and their half-sandwiched complexes have been prepared,^28−32^ no metal hydrides of bis-Cp ligands have been reported previously. For the bis-Cp ligand designation, we have opted for a 1,3-bis(dimethylsilyl)phenyl group as a spacer positioned between two bulky Cp^*t*Bu2^ donors to tightly hold metal centers. In addition, flexible sp^3^-hybridized SiMe_2_ linkages in the spacer are installed to adjust the distance between metal centers. In this work, we present the synthesis of the newly developed **H**_**2**_**L** (1,3-bis(2,4-di*-tert*-butylcyclopentadienyldimethylsilyl)benzene), along with its doubly deprotonated form, a bis-Cp ligand, **L**^**2–**^. Additionally, we detail the isolation and reactivity studies of an unprecedented lithium-stabilized di-iron trihydride complex (**LFe**_**2**_**(μ-H)**_**3**_**Li(THF)**; complex **2**) of **L**^**2–**^. This complex serves as a building block for constructing heterobimetallic complexes **4**–**6** with coinage metals.

## Results and Discussion

**Figure 1:**
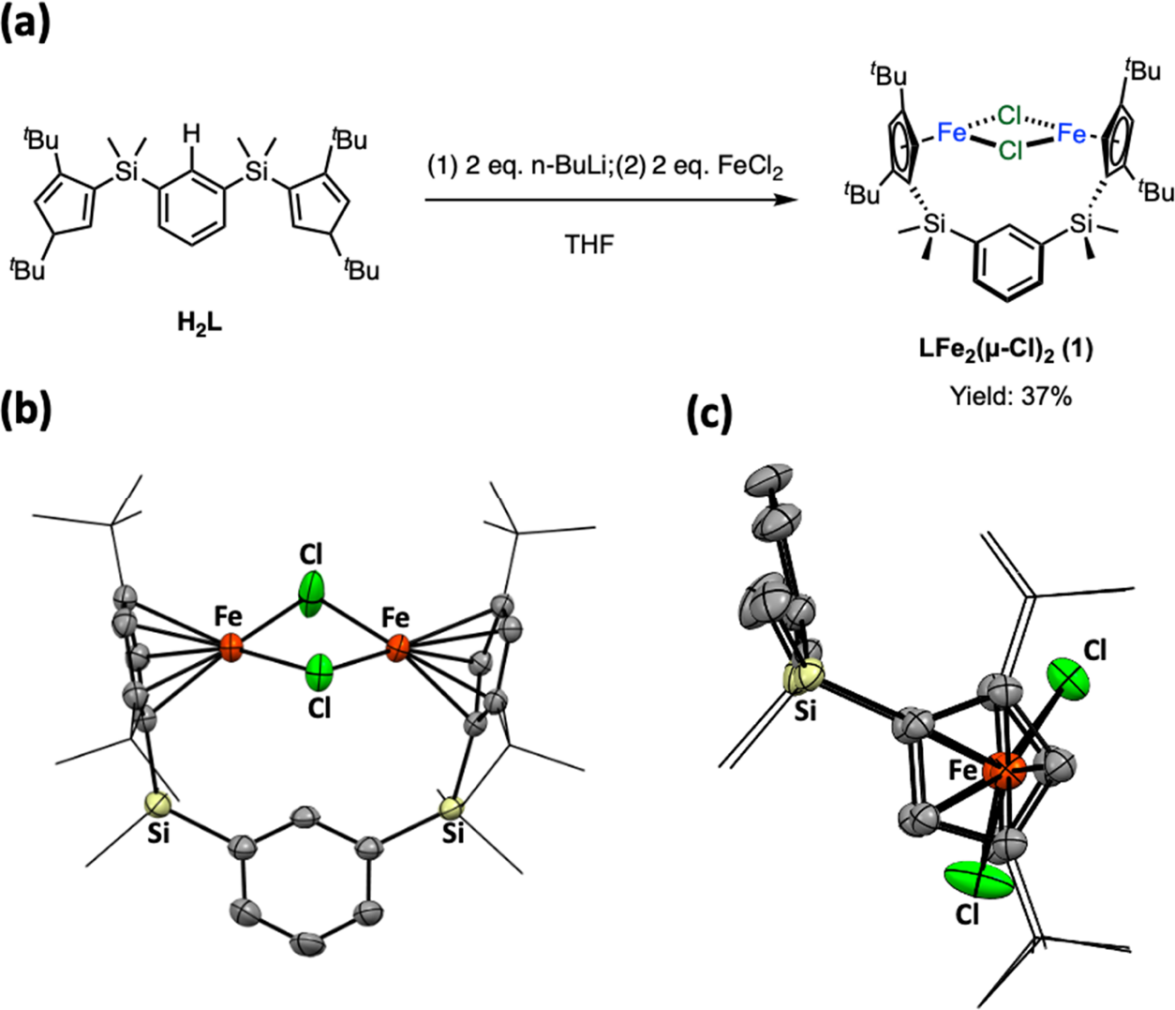
(a) Synthesis of complex **1**. (b) Front view and (c) side view of X-ray structure of complex **1**.

Having the designed ligand **H**_**2**_**L** in hand, the formation of a di-iron complex, **LFe**_**2**_**(μ-Cl)**_**2**_ (**1**), can be achieved by the addition of FeCl_2_ to the in situ prepared **Li**_**2**_**L** in THF, as illustrated in Figure 1. The slow evaporation of a saturated pentane solution of **1** resulted in the production of orange-yellow crystals with a yield of 37%. The X-ray diffraction analysis of complex **1** unveiled a butterfly-shaped and dimeric structure bridged by chloro ligands, with Fe–Cl bond lengths ranging from 2.3382(7) to 2.3559(6) Å and an Fe···Fe distance of 3.234 Å. The fold angle between two FeCl_2_ planes along a line through two Cl atoms is 27.9°, and the interplanar angle between two Cp^*t*Bu2^ rings in **1** is 22.11°. The average Fe–Cp_centroid_^*t*Bu2^ distance of 1.917 Å closely resembles that found in the high-spin [Cp′FeI]_2_ complex (1.93 Å), indicating the presence of two high-spin (*S* = 2) Fe(II) centers in **1**.^33^ The solution magnetic moment, determined by the Evans method, is 8.5 μB, corresponding to *S*_total_ = 4.

**Figure 2:**
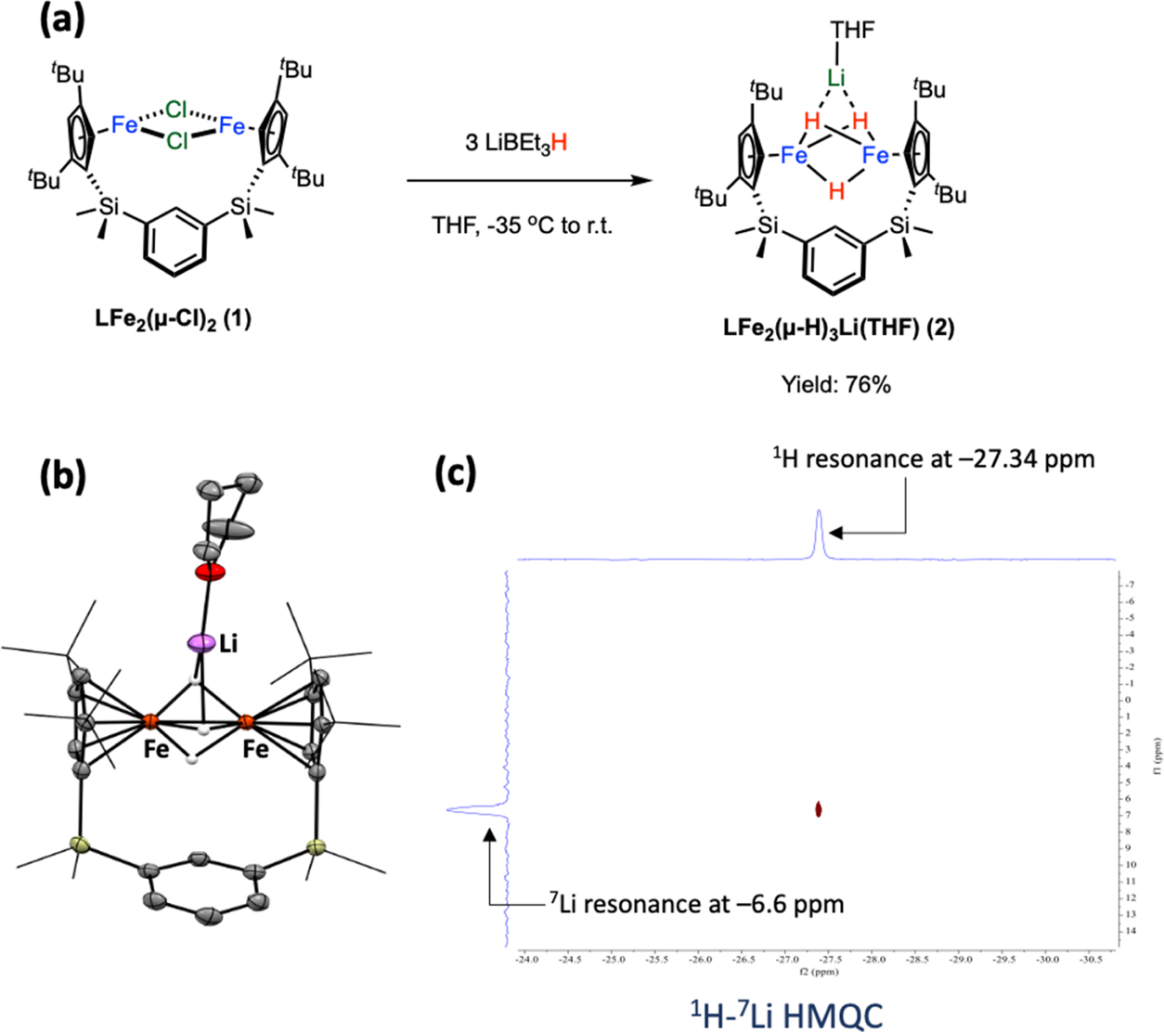
(a) Synthesis of complex **2**. (b) X-ray structure and (c) ^1^H–^7^Li HMQC NMR spectrum of complex **2**.

The reaction of complex **1** with 3 equiv of LiBEt_3_H in THF resulted in a rapid color change from orange to dark blue. Subsequent workup and crystallization enabled the isolation of **LFe**_**2**_**(μ-H)**_**3**_**Li(THF)** (**2**) as dark purple crystals, with a yield of 76%. The molecular structure of complex **2**, determined by single-crystal X-ray diffraction, revealed that two Cp^*t*Bu2^Fe moieties were closely held by three bridging hydride ligands, displaying an Fe···Fe distance of 2.2391(5) Å (see Figure 2b). Three hydrides, located in the difference map, exhibited Fe–H contacts within the range of 1.601–1.724 Å, typical for di-iron complexes with bridging hydride ligands.^20,21^ The Fe···Fe contact in complex **2** is slightly longer than those reported in tetra-hydride-bridged complexes **B**–**E**. Additionally, two of three hydrides also serve as bridging ligands between iron centers and the Li(THF) fragment with an average Li–H distance of 2.077 Å, thus resulting in short Fe···Li contacts of 2.571(7) Å—slightly shorter than the sum of covalent radii of Fe and Li (∼2.60 Å; for low-spin Fe).^34^ Complex **2** thus can be viewed as an anionic Cp_2_Fe_2_(μ-H)_3_ moiety stabilized by a cationic Li(THF) fragment. The contraction of the Fe···Fe distance by approximately 30% from **1** to **2**, along with a reduction in the distance between the Cp centroid in each Cp fragment to 5.561 from 6.943 Å (∼20%) from **1** to **2**, underscores the adaptability of **L** in supporting various binding interactions between two metal centers. To our knowledge, the anionic [(Cp^R^Fe)_2_(μ-H)_3_]^−^ moiety has so far been elusive and represents a novel type of CpFe hydrido complexes.

**Figure 3:**
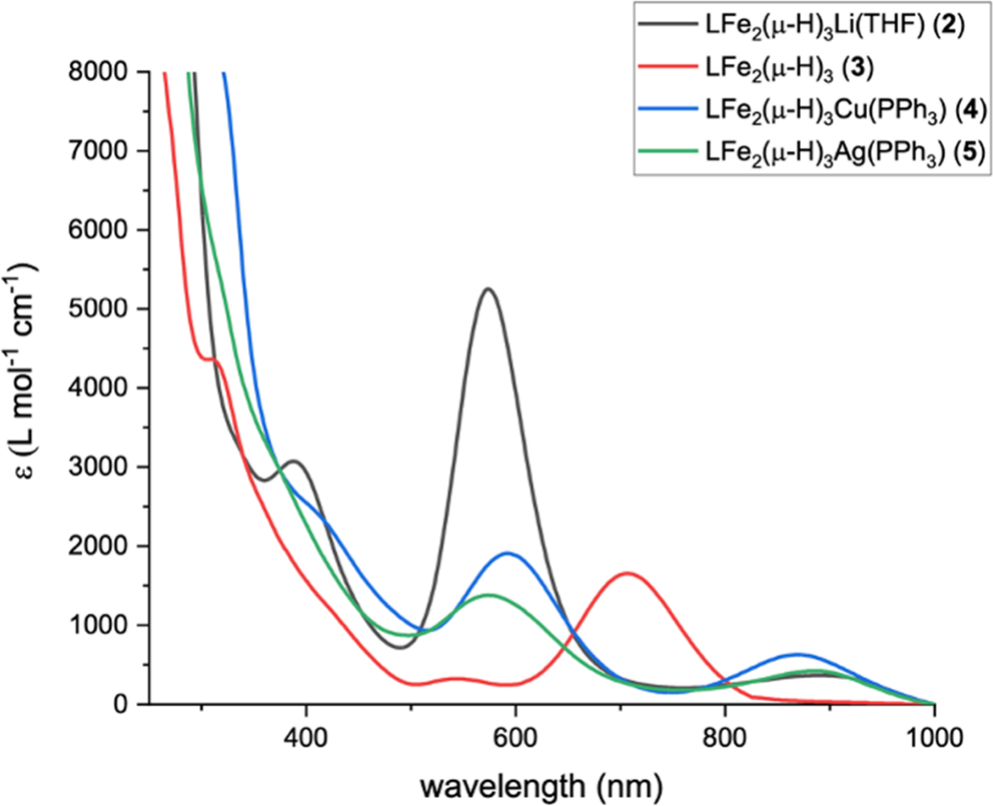
UV–vis spectra of complexes **2**–**5** in THF.

Complex **2** is diamagnetic and exhibits a sharp hydride resonance (3 H) at δ = −27.30 ppm in the ^1^H NMR spectrum at room temperature. The sharpness of this resonance persists even at −40 °C in *d*_8_-toluene, indicating the rapid fluxional behavior of the three hydride ligands. The hydride-lithium interaction can be probed by ^1^H–^7^Li HMQC NMR spectroscopy at −40 °C, where the hydride resonance correlates with the ^7^Li signal at δ 6.6 ppm (see Figure 2c), echoing the short Li–H distances found in the solid-state structure of **2**. The diamagnetic nature of complex **2** aligns with the short average Fe–Cp_centroid_^*t*Bu2^ distance of 1.653 Å in complex **2**, akin to those observed in low-spin and diamagnetic CpFe hydride complexes **B**–**E**. The UV–vis spectrum of complex **2** reveals three absorptions at 389 (ε = 3100), 573 (ε = 5300), and 896 (ε = 400) nm, with the intense absorption at 573 nm responsible for the dark-purple hue of complex **2** (see Figure 3).

The anionic nature of the {LFe_2_(μ-H)_3_} core of complex **2** suggests its potential as a reducing agent and a possible synthon for the **LFe**_**2**_**(μ-H)**_**3**_ core, allowing the isolation of heterometallic clusters forming Fe_2_H_3_M motifs. The emergence of heterobimetallic motifs in both homogeneous and heterogeneous systems has garnered attention due to their potential for activating bonds in small molecules and catalyzing reactions through bimetallic cooperation. Typically, these motifs are evaluated through reactions involving nucleophilic iron sites such as Fp (CpFe(CO)_2_^–^) with electrophilic coinage metal counterparts, as demonstrated, for instance, by Mankad.^35,36^ With complex **2** at our disposal, we aimed to explore its potential in synthesizing a novel class of iron-coinage heterobimetallic complexes.

**Figure 4:**
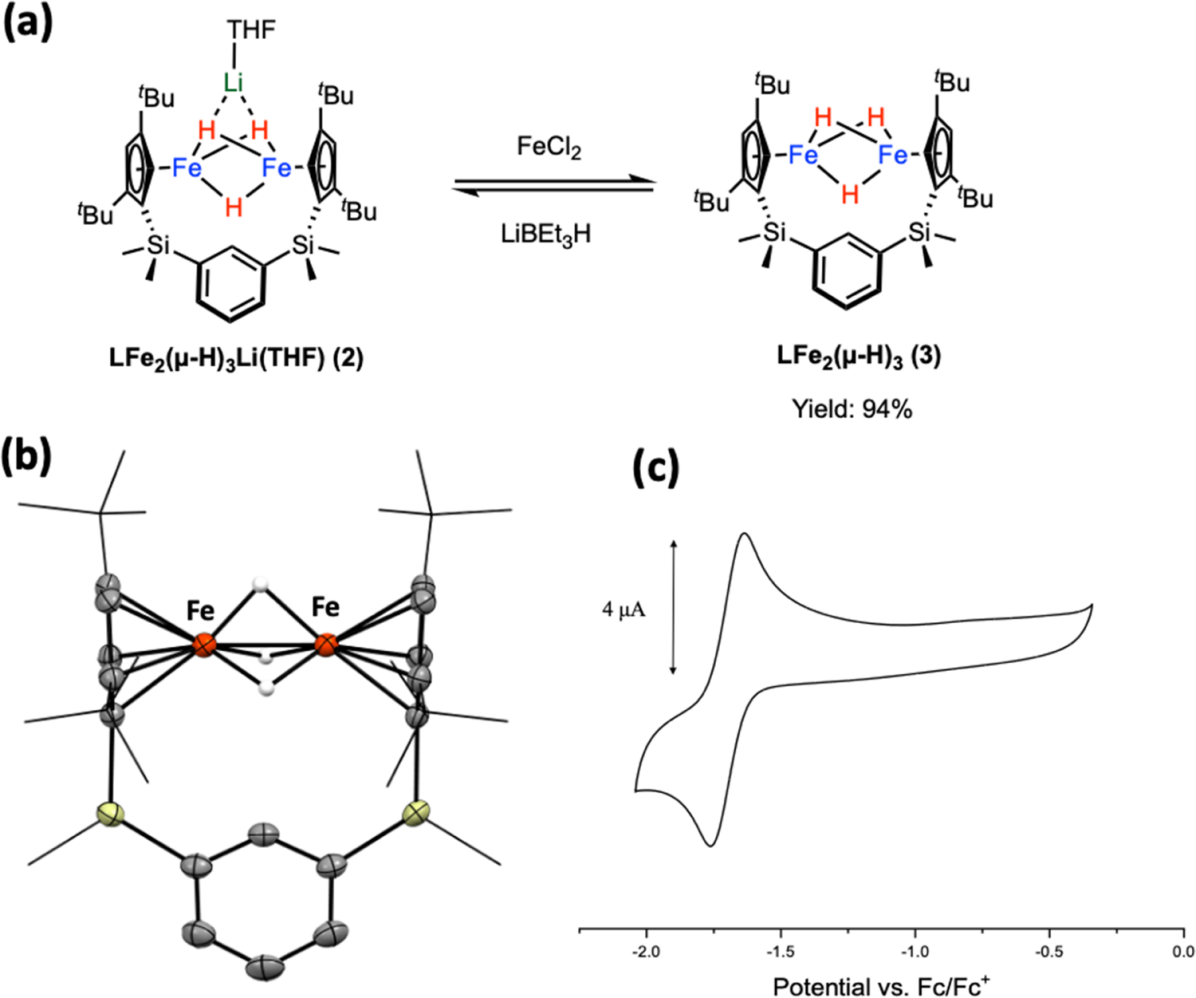
(a) Synthesis of complex **3**. (b) X-ray structure and (c) cyclic voltammogram of **3** (in 0.1 M THF solution of ^*n*^Bu_4_NPF_6_; scan rate = 100 mV s^–1^).

To investigate its redox behavior, we conducted cyclic voltammetry studies, which revealed a well-behaved reversible redox event at *E*_1/2_ = −1.73 V vs ferrocene/ferrocenium in THF (see Figure 4c). This negative redox potential indicates that complex **2** should be easily oxidized, thus prompting us to pursue the isolation and characterization of its oxidized congener. Reactions of complex **2** with metal halides such as FeCl_2_, CuCl_2_, or SnCl_2_ led to the oxidation of **2**, yielding a dark green complex **3** in high yields (see Figure 4a). Complex **3** is paramagnetic and exhibits effective magnetic moment μ_eff_ of 2.32 at 25 °C as determined by Evans method. The slightly higher μ_eff_ observed in complex **3** compared to the normal *S* = 1/2 complexes is likely due to the contribution of spin–orbit coupling. The single-crystal X-ray diffraction analysis of complex **3** reveals an Fe_2_H_3_ core supported by an **L**^**2–**^ framework with a slightly shorter Fe···Fe contact of 2.2232(14) Å compared to complex **2** (see Figure 4b**)**. The ^1^H NMR features of complex **3** are comparable to those observed in Walter’s Cp′Fe_2_(μ-H)_3_.^20^ The UV–vis absorption of complex **3** at 707 nm (ε = 1650 L mol^–1^ cm^–1^) is responsible for the dark green color (see Figure 3). The quantitative reverse reaction back to complex **2** can be observed when treating **3** with LiBEt_3_H. Treatment of **3** with other reducing agents such as Na, K, or KC_8_ resulted in **LFe**_**2**_**(μ-H)**_**3**_**M** (M = Na or K) with similar appearance and similar ^1^H NMR features to those of **2**. In addition, both complexes **2** and **3** are soluble in nonpolar solvents like benzene, toluene, and pentane. This solubility property is less common in redox couples and may find applications in synthetic chemistry involving redox events.

**Figure 5:**
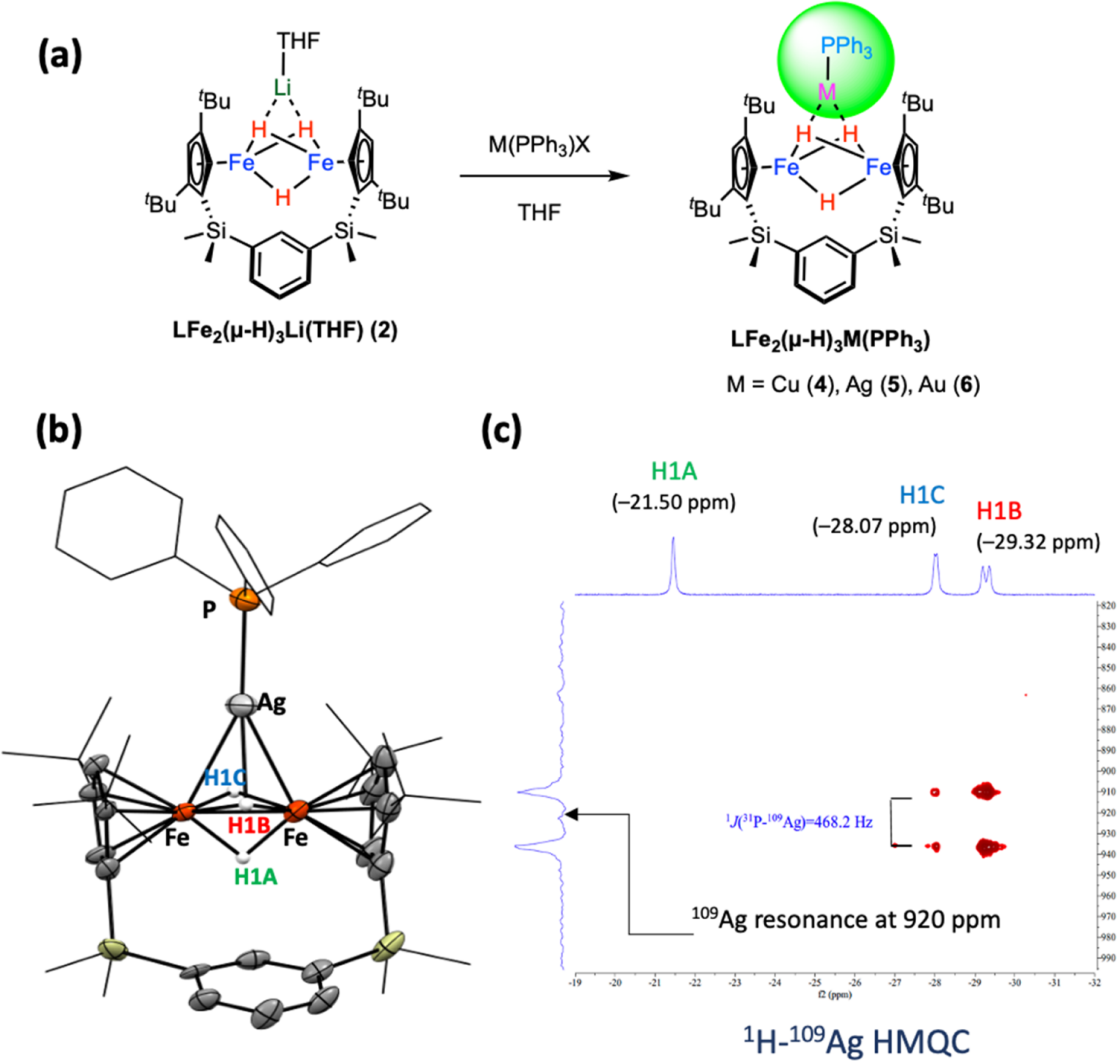
(a) Synthesis of complexes **4**–**6**. (b) X-ray structure and (c) ^1^H-^109^Ag HMQC NMR spectrum (toluene-*d*_8_, – 40 °C) of complex **5**.

The reactions with metal halides such as FeCl_2_ only led to oxidation of complex **2** to complex **3**, and no multimetallic species were identified. To address the potential electron transfer from **2** leading to its oxidation, metal salts supported by organic ligands were employed. Treatment of complex **2** with PPh_3_-stabilized coinage metal complexes cleanly afforded **LFe**_**2**_**(μ-H)**_**3**_**MPPh**_**3**_ (M = Cu (**4**), Ag (**5**), and Au (**6**); Figure 5a) in satisfactory to moderate yields. In all of these reactions, a rapid solution color change to dark brown-green was observed. The molecular structures of complexes **4**–**6** were determined by single-crystal X-ray diffraction experiments (see Figures 5b and S28 for **5**; Figure S27 for **4**; Figure S29 for **6**). All three complexes crystallize in a very similar unit cell in the *P*-1 space group, featuring an **Fe**_**2**_**H**_**3**_**M** core with close Fe···Fe contacts ranging from 2.25 to 2.27 Å. The average Fe–M distances of 2.5115(16) (**4**, M = Cu), 2.6900(17) (**5**, M = Ag), and 2.6701(12) (**6**, M = Au) are slightly shorter than the sum of the covalent radii of Fe and coinage metals,^34^ indicating minimal interactions between metal centers. Complexes **4**–**6** share a similar structure to complex **2**, wherein two hydrides act as bridging ligands between iron centers and MPPh_3_ fragments in all of these complexes. The selected bond lengths of complexes **2**–**6** are summarized in Table S1 in the SI.

**Figure 6:**
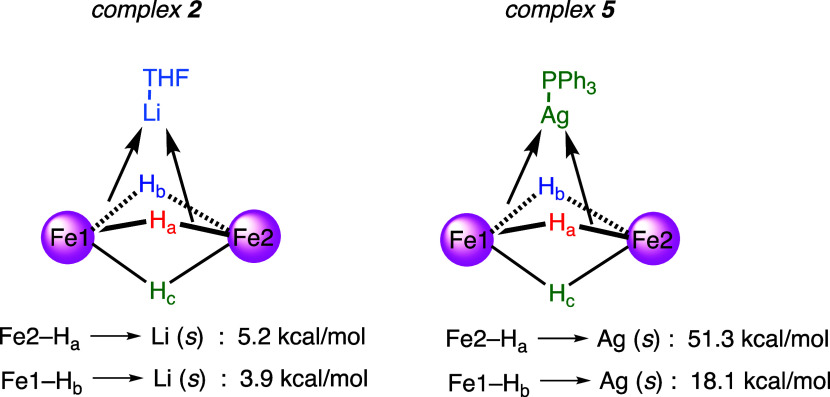
Donor–acceptor interactions in complexes **2** and **5** by second-order perturbation theory analysis in NBO.

In the ^1^H NMR spectra of complexes **4**–**6** in *d*_6_-benzene, the hydride ligands exhibit highly fluxional behavior, resulting in a broad resonance at 25 °C. Cooling down the *d*_8_-toluene solution of complexes **4** or **5** to −40 °C allows the observation of three nonequivalent hydride resonances with nearly equal intensity. For the ^1^H NMR spectrum of complex **5** (see Figure 5c), three resonances at −21.50 (singlet; H1A), −28.07 (doublet, ^2^*J*_HP_ = 20.2 Hz; H1C), and −29.32 ppm (doublet, ^2^*J*_HP_ = 63.2 Hz; H1B) show different degrees of interaction with the NMR-active phosphorus nuclei present on the AgPPh_3_ fragment. Additionally, the hydride resonance at −29.32 ppm shows a stronger correlation with the ^109^Ag resonance centered at 920 ppm as compared to the hydride at −28.07 ppm in the ^1^H-^109^Ag HMQC NMR spectrum (see Figure 5c). These NMR observations of **5** align with the Ag–H distances (Ag–H1B bond length of 2.269 Å and Ag–H1C bond length of 2.497 Å; see Figure S28) found in the crystal structure, suggesting significant hydride interactions with the AgPPh_3_ fragment. The spatial relationship between the three hydrides and the central phenyl proton was probed through one-dimensional nuclear Overhauser effect spectroscopy (NOSEY) at −40 °C, as illustrated in Figure S16 in the SI. Moreover, complexes **4** and **5** exhibit absorptions similar to complex **2** (see Figure 3). The bonding interactions between the heterometallic fragment and the Fe_2_(μ-H)_3_ core in complexes **2** and **5** were elucidated by natural bond orbital analysis (NBO). No direct metal–metal bonding was found, and two Fe–H bonds donating to Lewis acidic Li(THF) or Ag(PPh_3_) fragment (see Figure 6), together with the Coulomb forces between the cationic heterometallic groups and the anionic Fe_2_(μ-H)_3_ core, likely serve as main interactions that stabilize trimetallic clusters in complexes **2** and **5**. NBO results align well with the X-ray and NMR data of complex **5**, showcasing different degrees of Fe–H interactions with the Lewis acidic AgPPh_3_. In addition, significantly weaker interactions from Fe–H to the Lewis acidic fragment in complex **2** are observed compared to those in **5**, in agreement with the highly fluxional behavior of hydride ligands in complex **2** even at −40 °C as probed by ^1^H NMR and two-dimensional exchange NMR spectroscopy (EXSY; see Figure 17 in the SI).

## Conclusions

In summary, the newly synthesized bis-cyclopentadienyl ligand **L** readily affords well-defined binding pockets capable of accommodating diverse metal–ligand bonding interactions within the di-iron center. This capability allows for the isolation of the unprecedented **LFe**_**2**_**(μ-H)**_**3**_**Li(THF)** complex **2** with anionic nature and facilitates access to a series of heterobimetallic complexes. Ongoing investigations involve reactivity studies focused on bimetallic complex **2** and trinuclear complexes **4**–**6**, with the aim of further understanding and elucidating the metal–metal cooperation within these systems.

## Experimental Section

### General Information

All manipulations with oxygen and moisture-sensitive materials were performed in a nitrogen-filled glovebox. Solvents were dried and deaerated using a solvent system (AsiaWong Enterprise Co., Ltd.) prior to use. Benzene-*d*_6_ and tetrahydrofuran-*d*_8_ (THF-*d*_8_) were dried over sodium and benzophenone, degassed by three freeze–pump–thaw cycles, and stored under nitrogen over 3 Å molecular sieves. The NMR spectra were recorded using Bruker 300, Varian 400, or Varian 600 MHz spectrometer. The NMR spectra were referenced to residual protonated solvent for ^1^H NMR (3.58 ppm for compound in THF-*d*_8_; 7.16 ppm for compound in benzene-*d*_6_), to deuterated solvent for ^13^C NMR (67.21 ppm for compound in THF-*d*_8_ and 128.06 for compound in benzene-*d*_6_). All spectra were recorded at 25 °C. Complex multiplets are noted as “m” and broad resonances as “br”. Elemental analyses were performed using an Elementar Vario EL CUBE (CHN-OS Rapid, Germany). Mass spectrometry was performed using JMS-T200GC AccuTOF GCx (source mode: FD (field desorption)). Di-*tert*-butylcyclopentadiene (Cp^*t*Bu2^H) and its deprotonated form Cp^*t*Bu2^Li,^37^ m-Ph(SiMe_2_Cl)_2_,^38^ Cu(PPh_3_)Cl,^39^ Ag(PPh_3_)(OTf),^40^ and Au(PPh_3_)Cl^41^ were synthesized according to the reported procedures. Great attention must be exercised when working with pyrophoric chemicals like *n*-BuLi and LiBEt_3_H. The experiments were carried out using these substances under inert conditions, employing the Schlenk technique or gloveboxes.

### Synthesis of All Compounds

#### Synthesis of H_2_L

A THF solution (20 mL) containing Cp^*t*Bu2^Li (3.65 g, 19.8 mmol) was slowly added to a solution of m-Ph(SiMe_2_Cl)_2_ (2.55 g, 9.6 mmol) in THF (30 mL) at −35 °C. After stirring at ambient temperature for 14 h, the volatile components were removed in vacuo, and the product was extracted with hexane (100 mL), and the hexane was removed under reduced pressure, yielding **H**_**2**_**L** as a clear yellow oil (4.99 g, 95%) composed of a mixture of isomers. ^**1**^**H NMR** (400 MHz, benzene-*d*_6_, 298 K): δ = 7.96 (s, 1H, PhH), 7.52 (d, *J* = 7.7 Hz, 2H, PhH), 7.26 (t, *J* = 7.4 Hz, 1H, PhH), 6.55 (s, 2H, CpH), 5.91 (br, 2H, THF-α-CH_2_), 3.61 (br, 2H, CpH), 1.20 (s, 36H, ^*t*^Bu), 0.31 (s, 12H, SiMe_2_). ^**13**^**C{**^**1**^**H} NMR** (100 MHz, benzene-*d*_6_, 298 K): δ = 139.8, 139.0, 134.1, 128.1, 127.9, 127.5, 125.7, 33.2, 33.0, 31.3, −2.4 ppm. High-resolution field desorption mass spectrometry (HR-FD MS) calcd for C_36_H_58_Si_2_: 546.4083. Found: 546.4077.

#### Synthesis of LFe_2_(μ-Cl)_2_ (**1**)

A solution of **H**_**2**_**L** (5.86 g, 10.7 mmol) in pentane (100 mL) was treated with *n*-BuLi (1.6 M in hexane,13.2 mL, 20.8 mmol) at room temperature in a glovebox, and the mixture was stirred for 16 h. After stirring, the volatile components were removed in vacuo as much as possible, and the resulting colorless, viscous oil product was treated with 70 mL of Et_2_O. The product Li_2_L was formed as a white solid (4.58 g, 61%) by cooling the solution at −35 °C for 16 h. A suspension of FeCl_2_ (0.826 g, 6.47 mmol) in 50 mL of THF was added to the Li_2_L solution in THF (40 mL) at −35 °C, and the mixture was stirred at room temperature for 18 h. After this period, the volatile components were removed in vacuo and the product was added with toluene (50 mL). The mixture was heated to 70 °C for 16 h. After heating, the mixture was filtered, and the volatile components were removed in vacuo. The resulting orange solid was extracted with 30 mL of pentane. Slow evaporation of the pentane solution at room temperature afforded orange-yellow hexagonal crystals **LFe**_**2**_**(μ-Cl)**_**2**_. Yield: 0.820 g, 37%. ^**1**^**H NMR** (400 MHz, benzene-*d*_6_, 298 K) δ = 63.36 (br, 2H), 34.51 (br, 6H, SiMe_2_), 4.39 (br, 18H, *^t^*Bu), −6.71 (br, 18H, *^t^*Bu), −23.03 (br, 1H), −31.19 (br, 1H), −32.65 (br, 2H), −39.28 (br, 6H, SiMe_2_) ppm. Anal. calcd for **LFe**_**2**_**(μ-Cl)**_**2**_**·THF**, C_40_H_64_O_1_Si_2_Cl_2_Fe_2_: C, 60.08; H, 8.07. Found: C, 59.99; H, 7.69.

#### Synthesis of LFe_2_(μ-H)_3_Li(THF) (**2**)

A solution of LiBEt_3_H (1 M in THF, 1.23 mL, 1.23 mmol) was added dropwise to a precooled solution containing **LFe**_**2**_**(μ-Cl)**_**2**_ (**1**) (299 mg, 0.41 mmol) in 10 mL of THF. The clear orange solution turned dark blue upon addition. The mixture was warmed to ambient temperature and stirred for 1 h, and the volatile components were removed in vacuo. The resulting dark blue solid was extracted with 10 mL of toluene. Cooling down the toluene solution at −35 °C afforded dark purple crystals **LFe**_**2**_**(μ-H)**_**3**_**Li(THF)**. Yield: 229 mg, 76%. ^**1**^**H NMR** (400 MHz, benzene-*d*_6_, 298 K): δ = 11.90 (s, 1H, PhH), 7.72 (d, *J* = 6.2 Hz, 2H, PhH), 7.34 (t, *J* = 6.2 Hz, 1H, PhH), 4.57 (s, 2H, CpH), 3.56 (m, 2H, THF-α-CH_2_), 3.02 (s, 2H, CpH), 1.56 (s, 18H, tBu), 1.36 (s, 18H, tBu), 1.26 (m, 2H, THF-β-CH_2_), 0.81 (s, 6H, SiMe_2_), 0.62 (s, 6H, SiMe_2_), −27.33 (s, 3H, FeH). ^**13**^**C{**^**1**^**H} NMR** (100 MHz, benzene-*d*_6_, 298 K): δ = 149.5, 139.3, 132.8, 125.5, 103.7, 97.0, 74.0, 69.4, 62.0, 55.2, 33.5, 32.7, 31.9, 30.0, 25.3, 2.1, 1.6 ppm. UV–vis: 573 nm (ε = 5250), 890 nm (ε = 370) in THF. Anal. calcd for **LFe**_**2**_**(μ-H)**_**3**_**Li(THF)·toluene**, C_47_H_75_O_1_Li_1_Si_2_Fe_2_: C, 67.94; H, 9.10. Found: C, 68.44; H, 9.10.

#### Synthesis of LFe_2_(μ-H)_3_ (**3**)

A suspension of FeCl_2_ (8.6 mg, 0.067 mmol) in 5 mL of THF was slowly added to a precooled THF solution (10 mL, −35 °C) of **LFe**_**2**_**(μ-H)**_**3**_**Li(THF)** (50 mg, 0.067 mmol). The clear purple solution turned dark green upon addition. The mixture was warmed to ambient temperature and stirred for 14 h, and the volatile components were removed in vacuo. The resulting dark green solid was extracted with 10 mL of toluene. Cooling down the concentrated toluene solution at −35 °C afforded dark green crystals **LFe**_**2**_**(μ-H)**_**3**_. Yield: 42 mg, 94%. ^**1**^**H NMR** (400 MHz, benzene-*d*_6_, 298 K) δ = 37.09 (br, 2H), 27.73 (br, 2H), 7.27 (br, 6H, SiMe_2_), −2.12 (br, 1H), −3.07 (br, 1H), −6.54 (br, 6H, SiMe_2_), −7.87 (br, 18H, ^*t*^Bu), −9.18 (br, 18H, *^t^*Bu), −66.54 (br, 1H) ppm. UV–vis: 545 nm (ε = 325), 705 nm (ε = 1650) in THF. Anal. calcd for **LFe**_**2**_**(μ-H)**_**3**_, C_36_H_59_Si_2_Fe_2_: C, 65.54; H, 9.01. Found: C, 65.98; H, 9.14.

#### Synthesis of LFe_2_(μ-H)_3_M(PPh_3_) (M = Cu (**4**), Ag (**5**) Au (**6**))

##### For the Synthesis of **4**

A solution of CuPPh_3_Cl (48.4 mg, 0.134 mmol) in 5 mL of toluene was slowly added to a precooled toluene solution (10 mL, −35 °C) of **LFe**_**2**_**(μ-H)**_**3**_**Li(THF)** (100 mg, 0.134 mmol). The clear purple solution turned dark green upon addition. The mixture was warmed to ambient temperature and stirred for 1 h, and the volatile components were removed in vacuo. The resulting dark green solid was extracted with 10 mL of pentane. Cooling down the concentrated toluene solution at −35 °C afforded dark green crystals **LFe**_**2**_**(μ-H)**_**3**_**Cu(PPh**_**3**_**)**. Yield: 98.7 mg, 74%.^**1**^**H NMR** (400 MHz, benzene-*d*_6_, 298 K): δ = 11.25 (s, 1H, PhH), 7.74 (t, *J* = 9.4 Hz, 6H, PPh_3_), 7.67 (d, *J* = 6.9 Hz, 2H, PhH), 7.26 (t, *J* = 8.0 Hz, 1H, PhH), 7.13–7.01 (m, 9H, PPh_3_), 5.07 (s, 2H, CpH), 4.04 (s, 2H, CpH), 1.52 (s, 18H, ^*t*^Bu), 1.33 (s, 18H, *^t^*Bu), 0.80 (s, 6H, SiMe_2_), 0.65 (s, 6H, SiMe_2_), −28.10 (br, 3H, FeH). ^**13**^**C{**^**1**^**H} NMR** (100 MHz, benzene-*d*_6_, 298 K): δ = 150.8, 139.1, 134.5, 134.4, 132.7, 132.2, 131.8, 130.8, 129.1, 129.0, 128.1, 127.9, 125.6, 34.7, 33.5, 31.1, 29.5, 2.6, −0.1 ppm. UV–vis: 590 nm (ε = 1900), 870 nm (ε = 630) in THF. Anal. calcd for **LFe**_**2**_**(μ-H)**_**3**_**Cu(PPh**_**3**_**)·toluene**, C_61_H_82_Si_2_P_1_Fe_2_Cu_1_: C, 67.98; H, 7.67. Found: C, 68.05; H, 7.84.

##### For the Synthesis of **5**

A similar procedure for the synthesis of **4** was applied, and AgPPh_3_OTf (69.6 mg, 0.134 mmol) was used. Yield: 75.3 mg, 54%.^**1**^**H NMR** (400 MHz, benzene-*d*_6_, 298 K): δ = 12.00 (s, 1H, PhH), 7.74 (t, *J* = 8.4 Hz, 6H, PPh_3_), 7.68 (d, *J* = 7.3 Hz, 2H, PhH), 7.30 (t, *J* = 7.1 Hz, 1H, PhH), 7.14–7.04 (m, 9H, PPh_3_), 4.89 (s, 2H, CpH), 4.12 (s, 2H, CpH), 1.50 (s, 18H, *^t^*Bu), 1.34 (s, 18H, *^t^*Bu), 0.88 (s, 6H, SiMe_2_), 0.58 (s, 6H, SiMe_2_), −26.59 (br, 3H, FeH). ^**13**^**C{**^**1**^**H} NMR** (100 MHz, benzene-*d*_6_, 298 K): δ = 149.3, 138.8, 134.1, 132.6, 132.3, 132.0, 130.9, 129.3, 129.3, 128.1, 127.9, 125.6, 105.1, 95.9, 73.5, 65.2, 57.7, 33.9, 33.0, 31.0, 29.5, 2.4, −1.3 ppm. UV–vis: 574 nm (ε = 1380), 887 nm (ε = 422) in THF. Anal. calcd for **LFe**_**2**_**(μ-H)**_**3**_**Ag(PPh**_**3**_**)**, C_54_H_74_P_1_Si_2_Fe_2_Ag_1_: C, 62.98; H, 7.24. Found: C, 62.64; H, 7.52.

##### For the Synthesis of **6**

A similar procedure for the synthesis of **4** was applied, and AuPPh_3_Cl (66.3 mg, 0.134 mmol) was used. Yield: 68.2 mg, 45%.^**1**^**H NMR** (400 MHz, benzene-*d*_6_, 298 K): δ = 9.07 (s, 1H, PhH), 7.57 (m, 6H, PPh_3_), 7.49 (d, *J* = 7.2 Hz, 2H, PhH), 7.13 (t, *J* = 7.3 Hz, 1H, PhH), 7.07–6.98 (m, 9H, PPh_3_), 5.90 (s, 2H, CpH), 5.72 (s, 2H, CpH), 1.44 (s, 18H, *^t^*Bu), 1.34 (s, 18H, ^*t*^Bu), 0.81 (s, 6H, SiMe_2_), 0.72 (s, 6H, SiMe_2_), −32.72 (br, 3H, FeH). ^**13**^**C{**^**1**^**H} NMR** (100 MHz, benzene-*d*_6_, 298 K): δ = 152.1, 139.5, 134.3, 133.3, 133.0, 132.6, 130.8, 129.3, 128.8, 128.5, 128.1, 127.9, 125.5, 36.5, 35.2, 31.5, 29.0, 2.5, 2.4 ppm. Due to its thermal instability, no satisfactory elemental analysis results were obtained.

## Supporting Information Available

General considerations, synthetic procedures, compound characterizations, theoretical calculation details, and crystallographic data for all compounds (PDF)
