# Supplementary material for: A Bis-Cyclopentadienyl Ligand-Supported Di-Iron Trihydride Motif as a Synthon for Access to Heterobimetallic Trinuclear Complexes
Source: Inorg Chem. 2024 May 30;63(24):11361–8. doi: 10.1021/acs.inorgchem.4c01420 (PMC11190976; doi:10.1021/acs.inorgchem.4c01420)
Supplement: Supplementary file 1 — ic4c01420_si_001.pdf [file ic4c01420_si_001.pdf]

Supporting Information

A Bis-Cyclopentadienyl Ligand Supported Di-Iron Trihydride Motif as a Synthon for Access to Heterobimetallic Trinuclear Complexes

Chung-Ching Tseng,[a],† Yi-Wun Ding,[a],† Zhong-Yue Chen,[a] Hao-Yuan Lan,[a] Han-Jung Li,[a] You-Song Cheng,[a] Ting-Shen Kuo,[c] Pei-Lin Chen,[d] Wen-Chun Wu,[e] Fong-Ku Shi,[e] Tzuhsiung Yang,[d] and Hsueh-Ju Liu*[a][b]

[a] Department of Applied Chemistry, National Yang Ming Chiao Tung University, Hsinchu City 300093, Taiwan

[b] Center for Emergent Functional Matter Science, National Yang Ming Chiao Tung University, Hsinchu City 300093, Taiwan

[c] Department of Chemistry, National Taiwan Normal University, Taipei 11677, Taiwan

[d] Department of Chemistry, National Tsing Hua University, Hsinchu city 300044, Taiwan

[e] Rezwave Technology Inc., 3F-5, 79, Hsin Tai Wu Rd., Sec.1, HsiChih District, New Taipei City, Taiwan

Corresponding author's E-mail: hsuehjuliu@nycu.edu.tw

Table of content:

1. NMR CHARACTERIZATIONS FOR ALL COMPOUNDS 2
2. UV-VIS SPECTRA FOR ALL COMPOUNDS 14
3. SINGLE-CRYSTAL X-RAY DIFFRACTION CRYSTAL STRUCTURE FIGURES AND DATA TABLES 16
4. IR SPECTRA 29
5. COMPUTATIONAL RESULTS 32
6. REFERENCES 33

1. NMR characterizations for all compounds

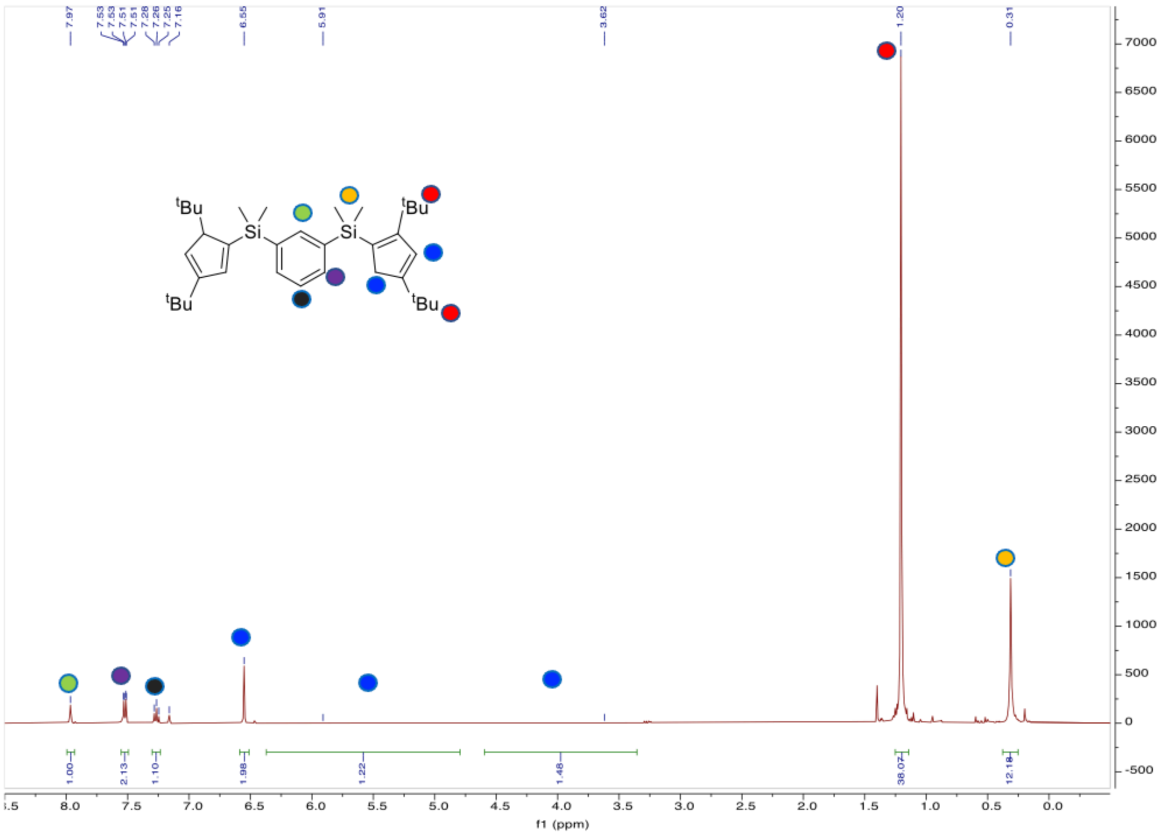

Figure S1. ^{1}H NMR spectrum of **H_2L** in C_6D_6.

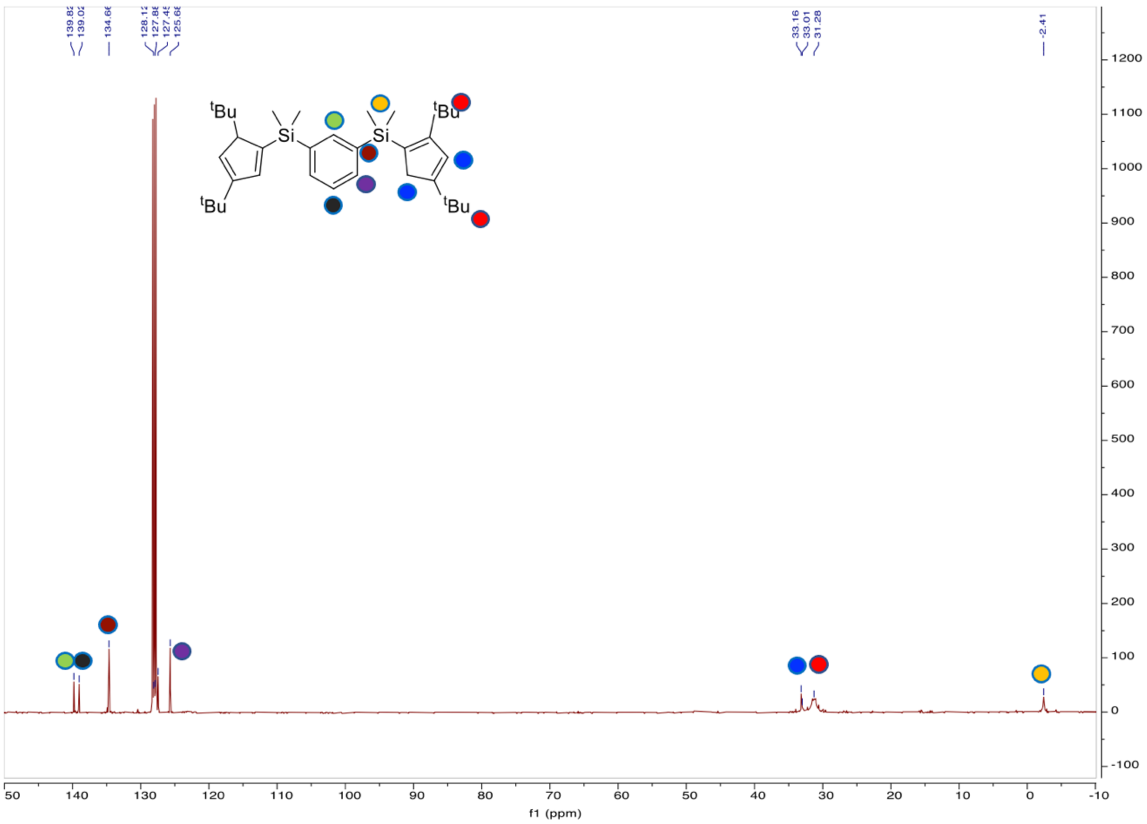

Figure S2. $^{13}C\{^1H\}$ NMR spectrum of **H_2L** in C_6D_6.

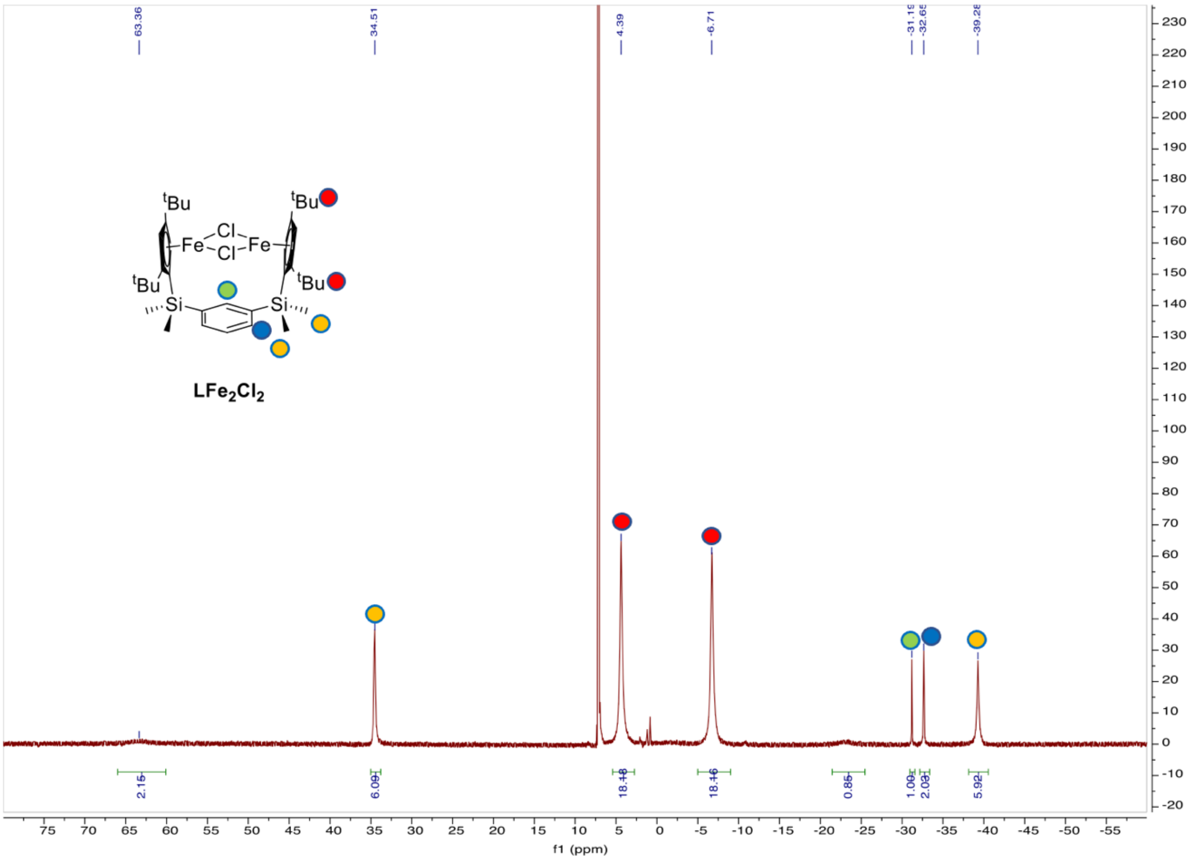

Figure S3. ^{1}H NMR spectrum of **$LFe_2(\mu\text{-}Cl)_2$ (1)** in C_6D_6.

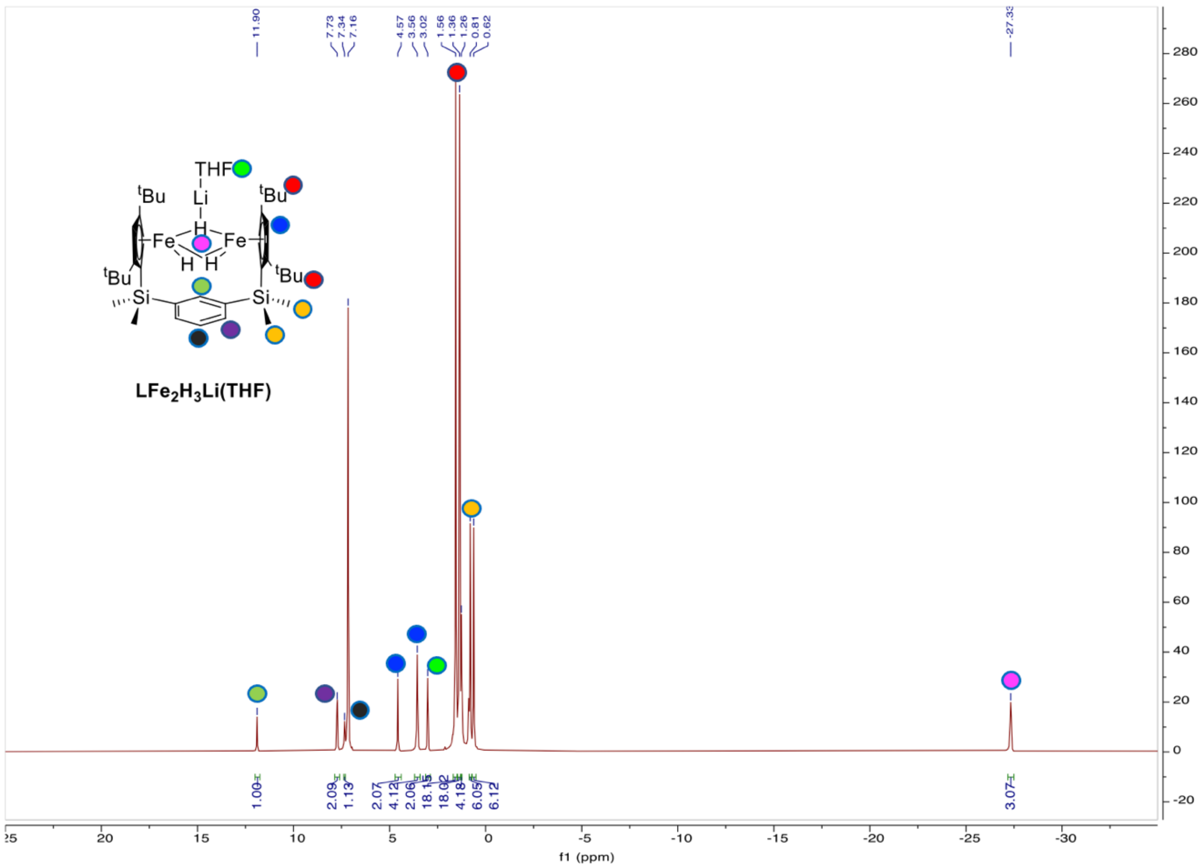

Figure S4. ^{1}H NMR spectrum of **$LFe_2(\mu\text{-}H)_3Li(THF)$ (2)** in C_6D_6.

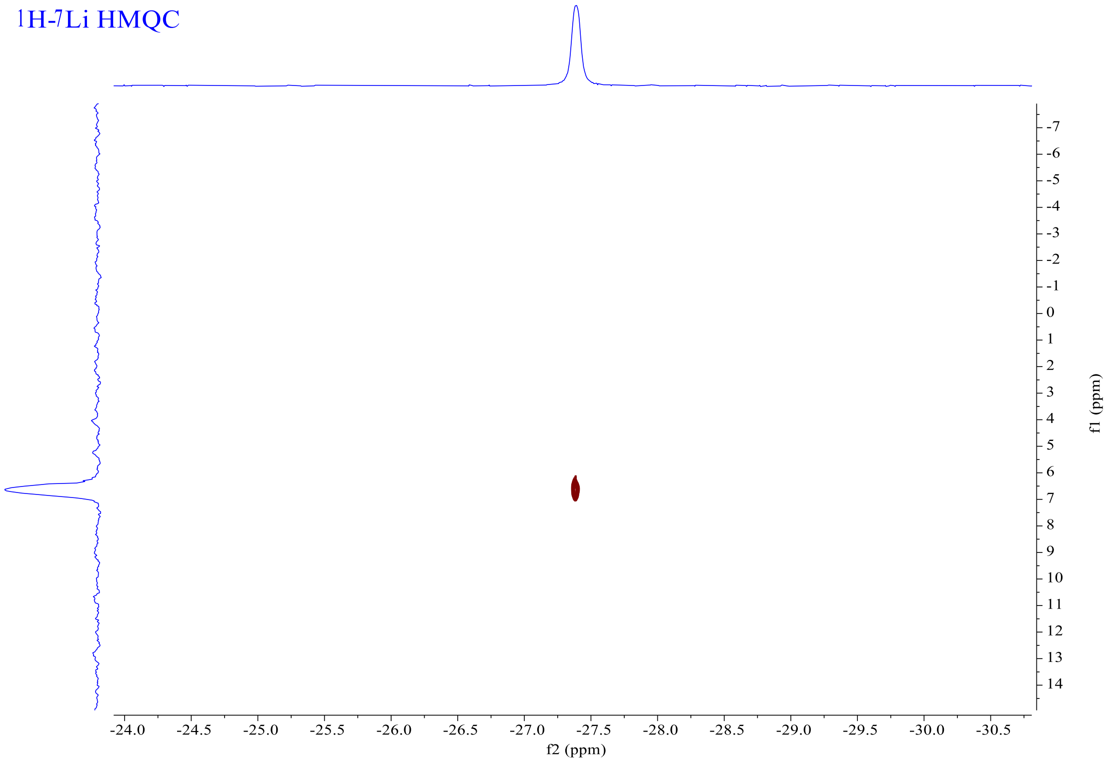

Figure S5. ^{1}H-^{7}Li HMQC NMR spectrum of **$LFe_2(\mu\text{-}H)_3Li(THF)$ (2)** in C_6D_6.

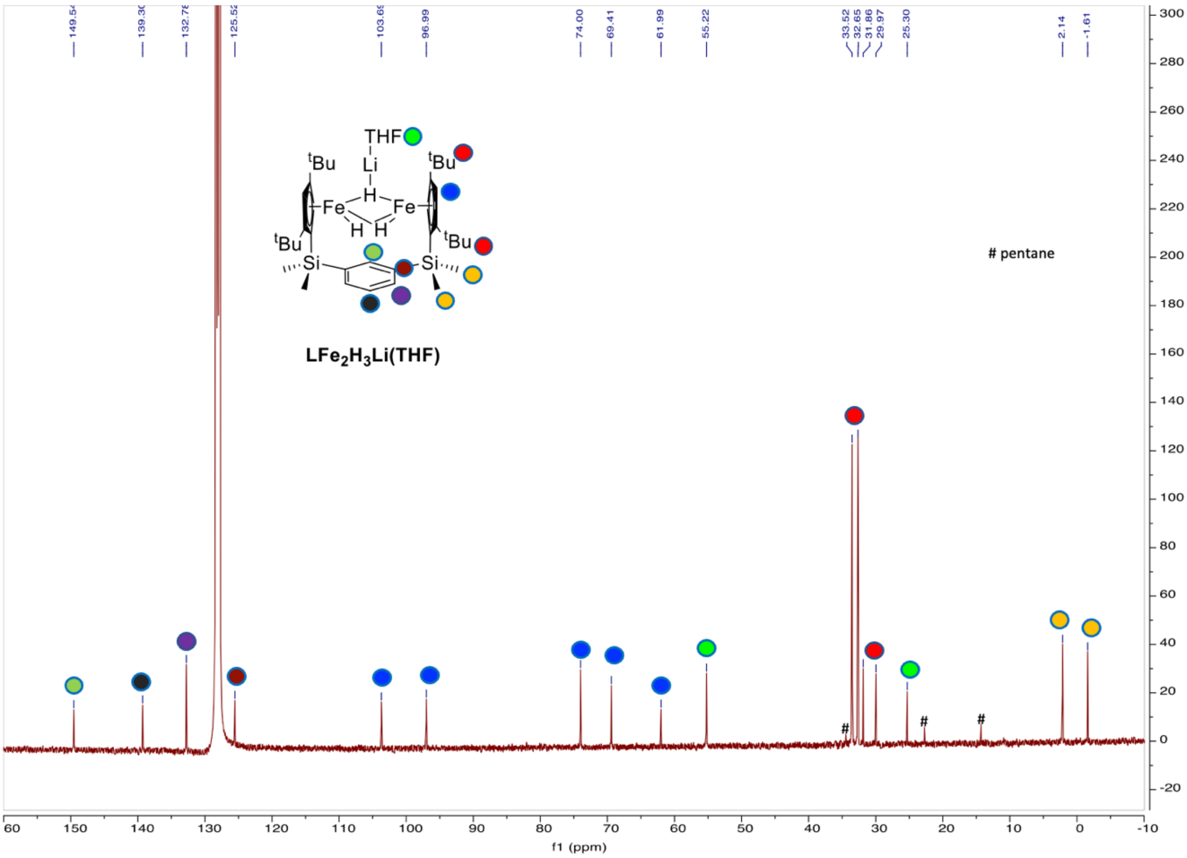

Figure S6. $^{13}C\{^1H\}$ NMR spectrum of **$LFe_2(\mu\text{-}H)_3Li(THF)$ (2)** in C_6D_6.

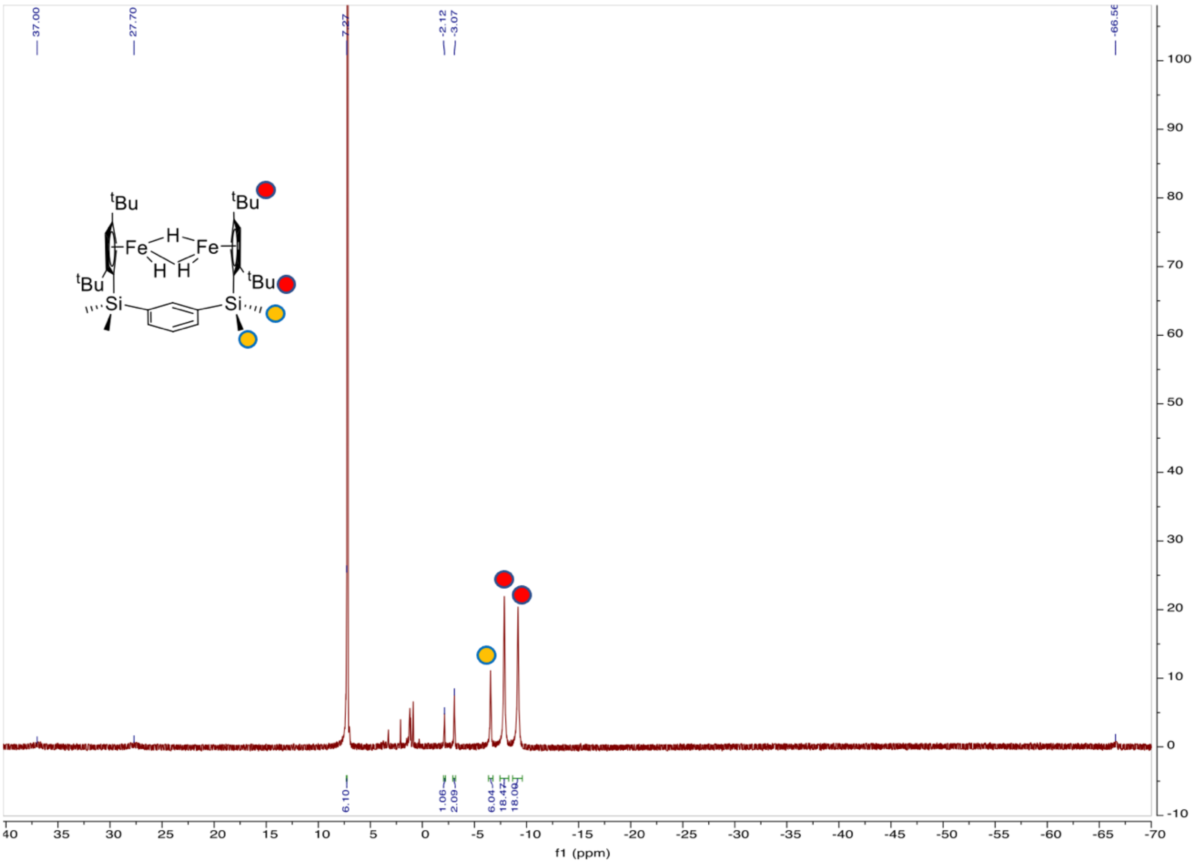

Figure S7. ^{1}H NMR spectrum of **$LFe_2(\mu$-$H)_3$ (3)** in C_6D_6.

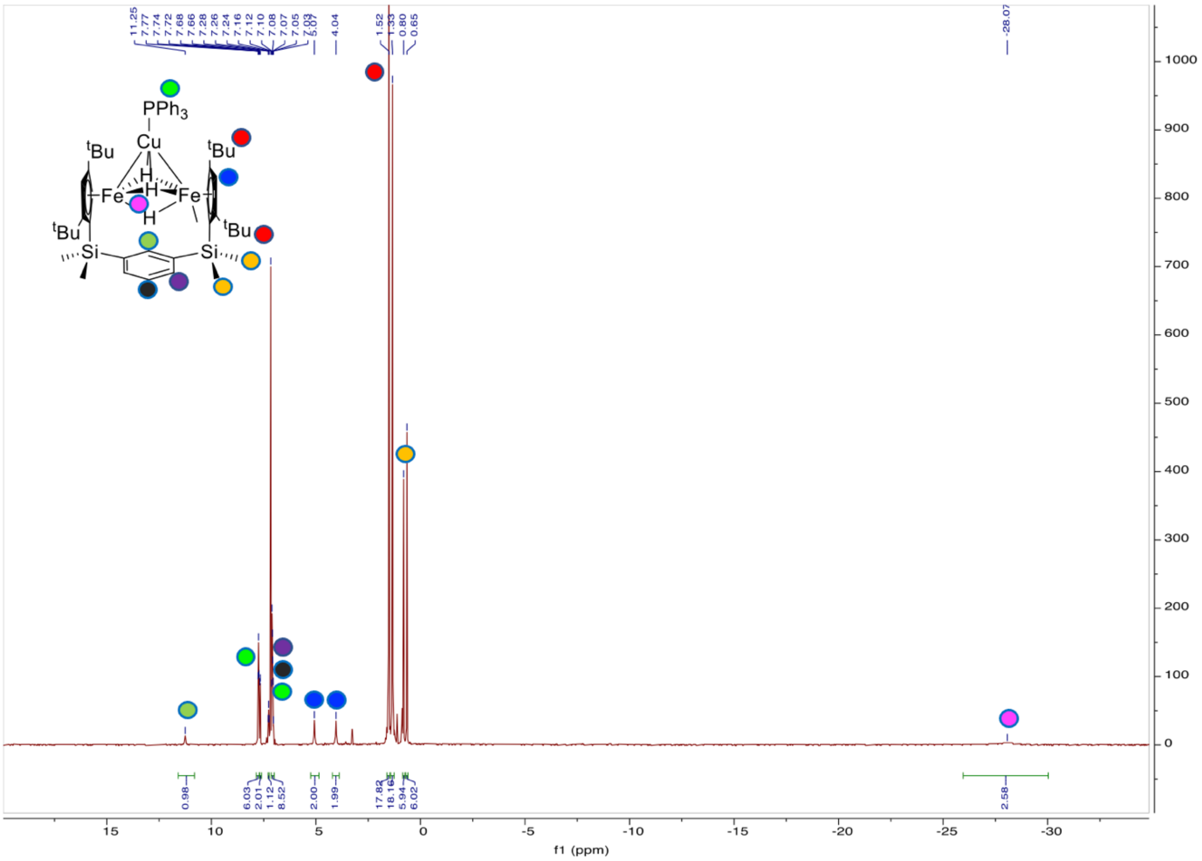

Figure S8. ^{1}H NMR spectrum of **LFe$_2$(μ-H)$_3$Cu(PPh$_3$) (4)** in C_6D_6.

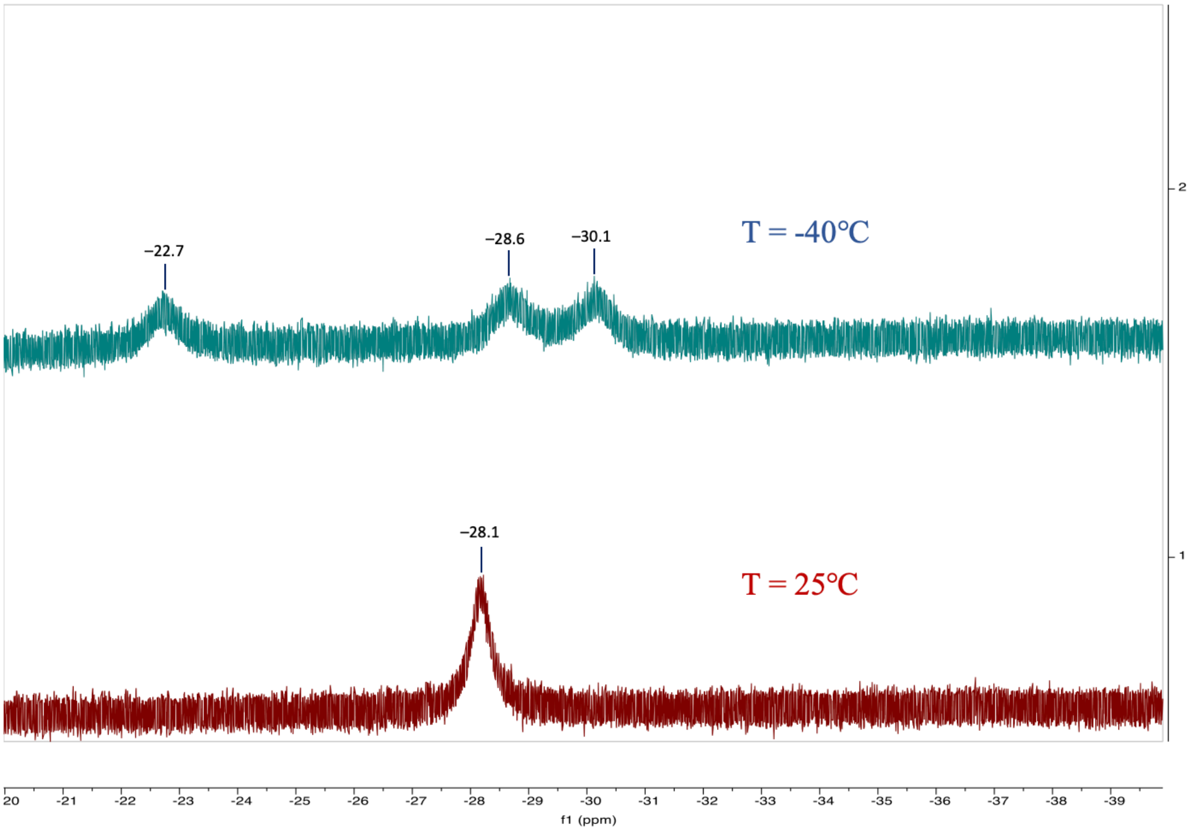

Figure S9. Partial ^{1}H NMR spectrum of **LFe$_2$(μ-H)$_3$Cu(PPh$_3$) (4)** in toluene-*d*$_8$ at –40 °C.

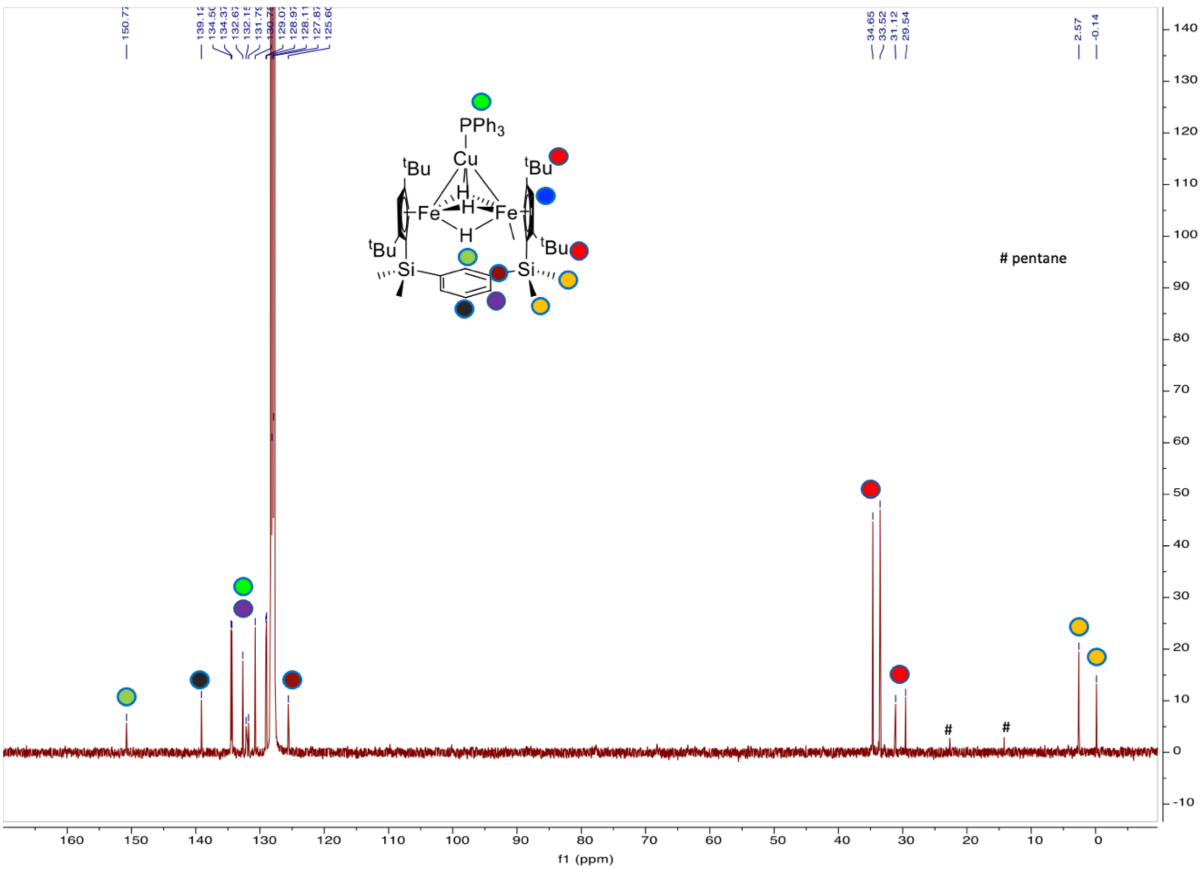

Figure S10. $^{13}C\{^1H\}$ NMR spectrum of **$LFe_2(\mu\text{-}H)_3Cu(PPh_3)$ (4)** in benzene-d_6.

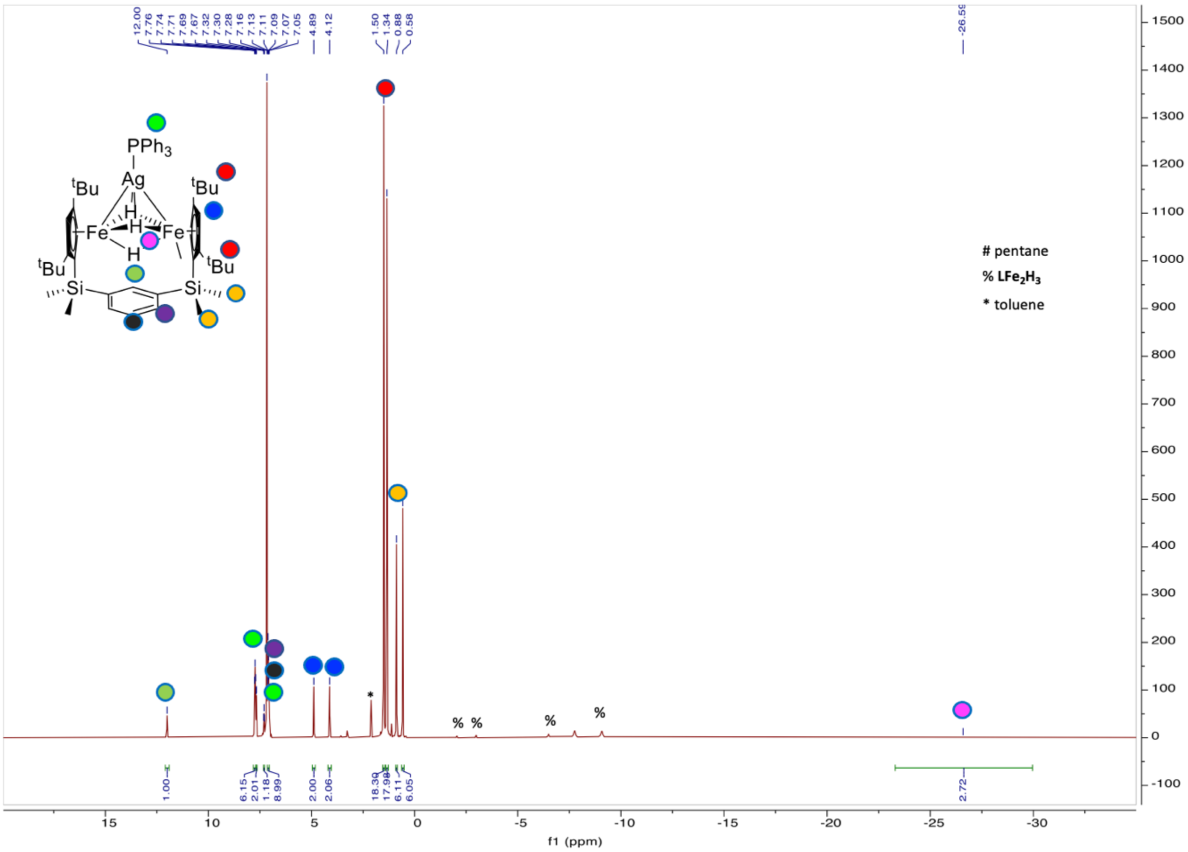

Figure S11. ^{1}H NMR spectrum of **LFe$_2$(μ-H)$_3$Ag(PPh$_3$) (5)** in benzene-d_6.

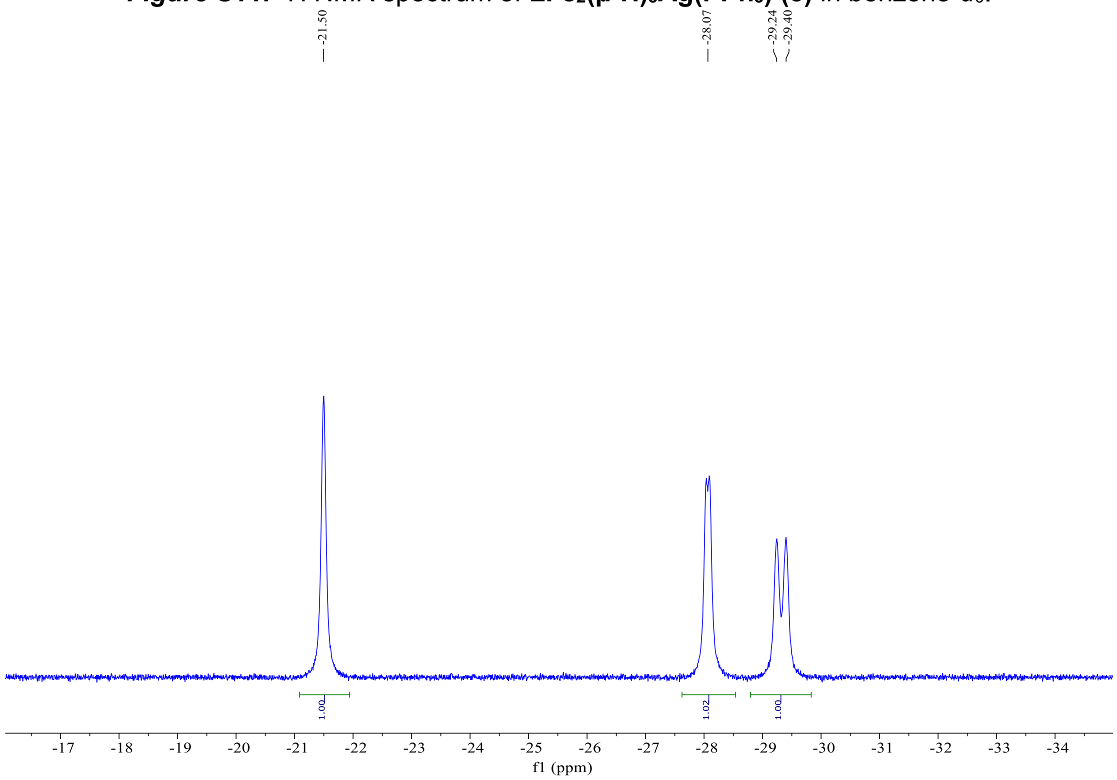

Figure S12. Partial ^{1}H (the hydride region) NMR spectrum of **LFe$_2$(μ-H)$_3$Ag(PPh$_3$) (5)** in toluene-d_8 at –40 °C.

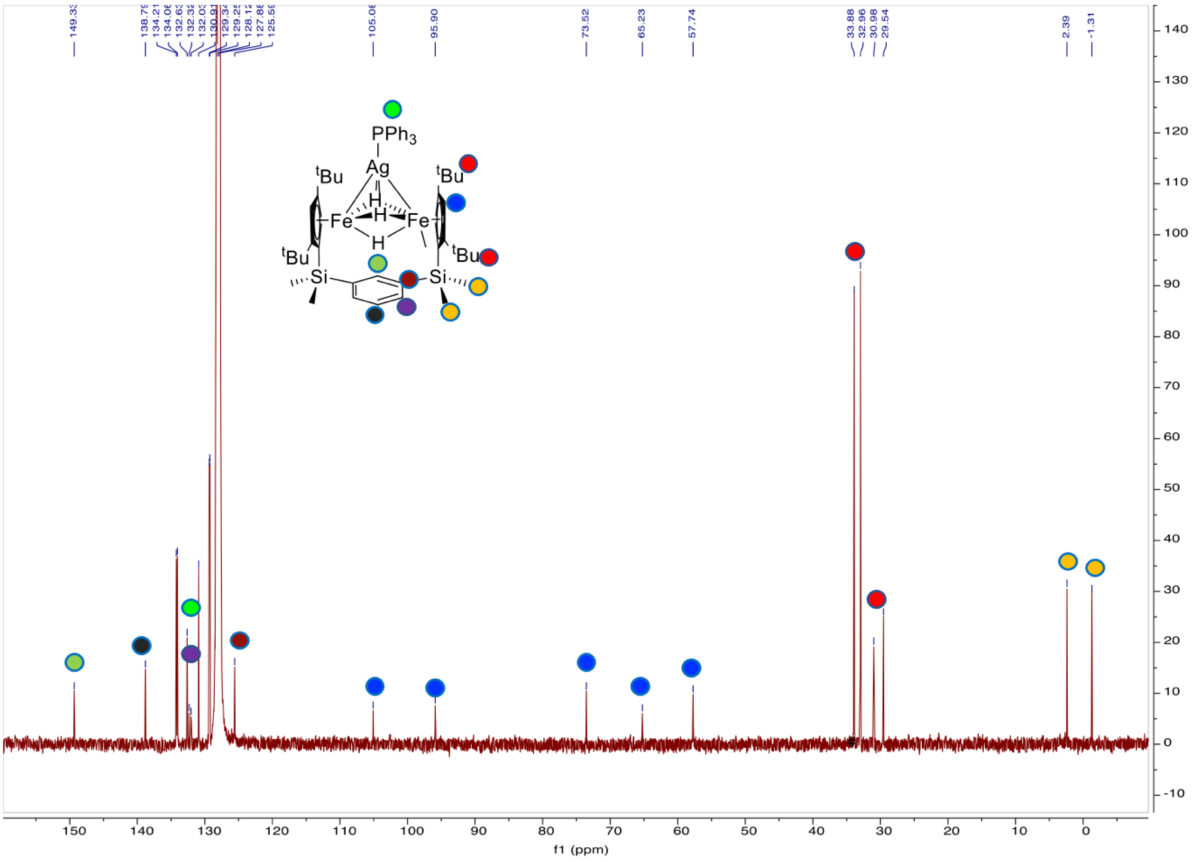

Figure S13. $^{13}C\{^{1}H\}$ NMR spectrum of **LFe$_2$(μ-H)$_3$Ag(PPh$_3$) (5)** in benzene-d_6.

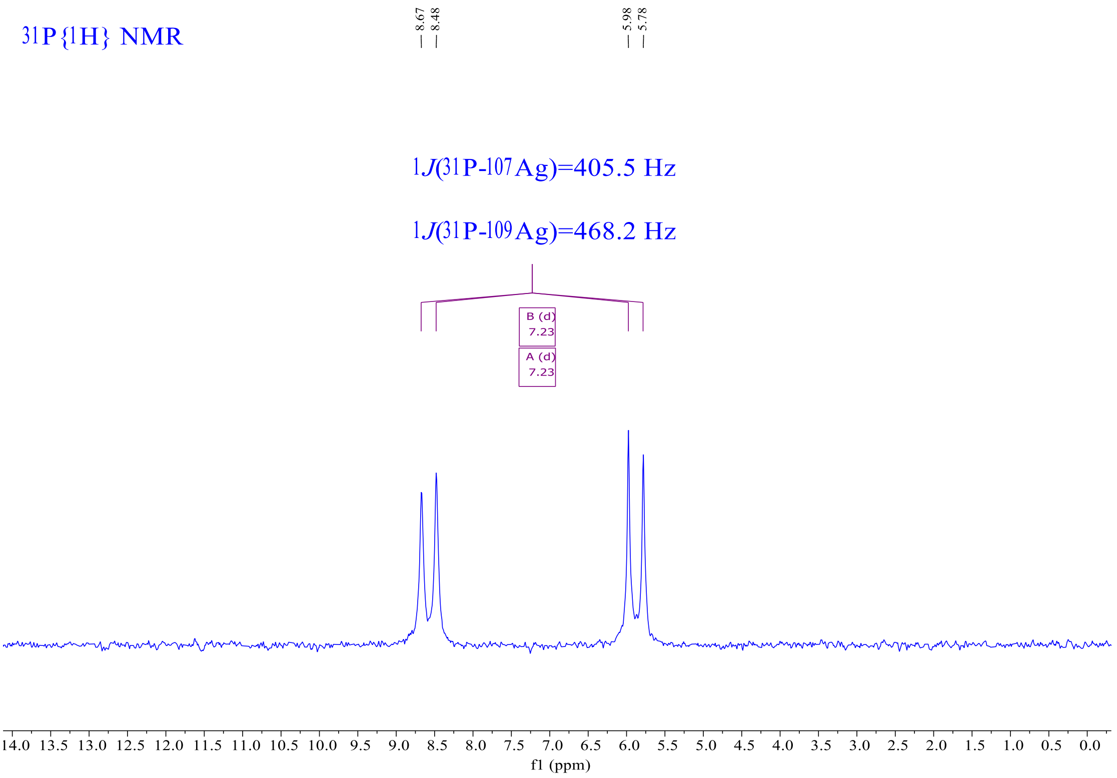

Figure S14. ^{31}P NMR spectrum of **LFe$_2$(μ-H)$_3$Ag(PPh$_3$) (5)** in toluene-d_8.

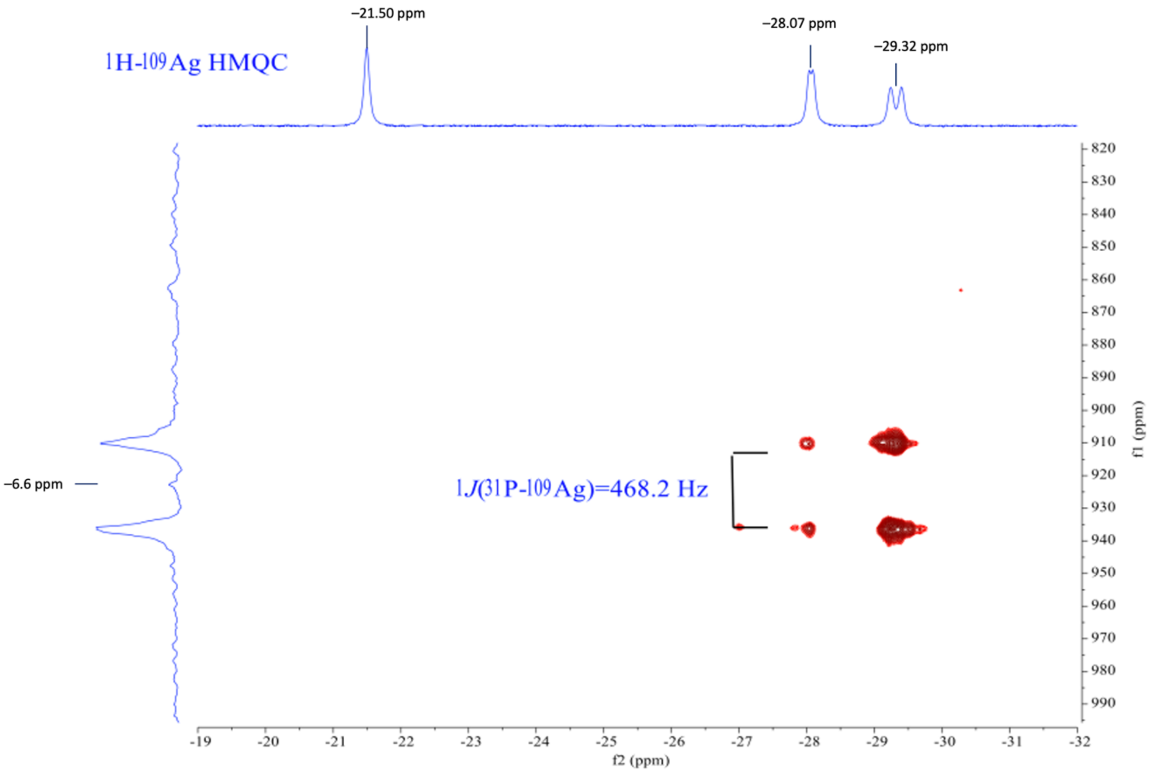

Figure S15. ^1H-^{109}Ag HMQC NMR spectrum of **LFe₂(μ-H)₃Ag(PPh₃) (5)** in toluene-d_8 at –40 °C.

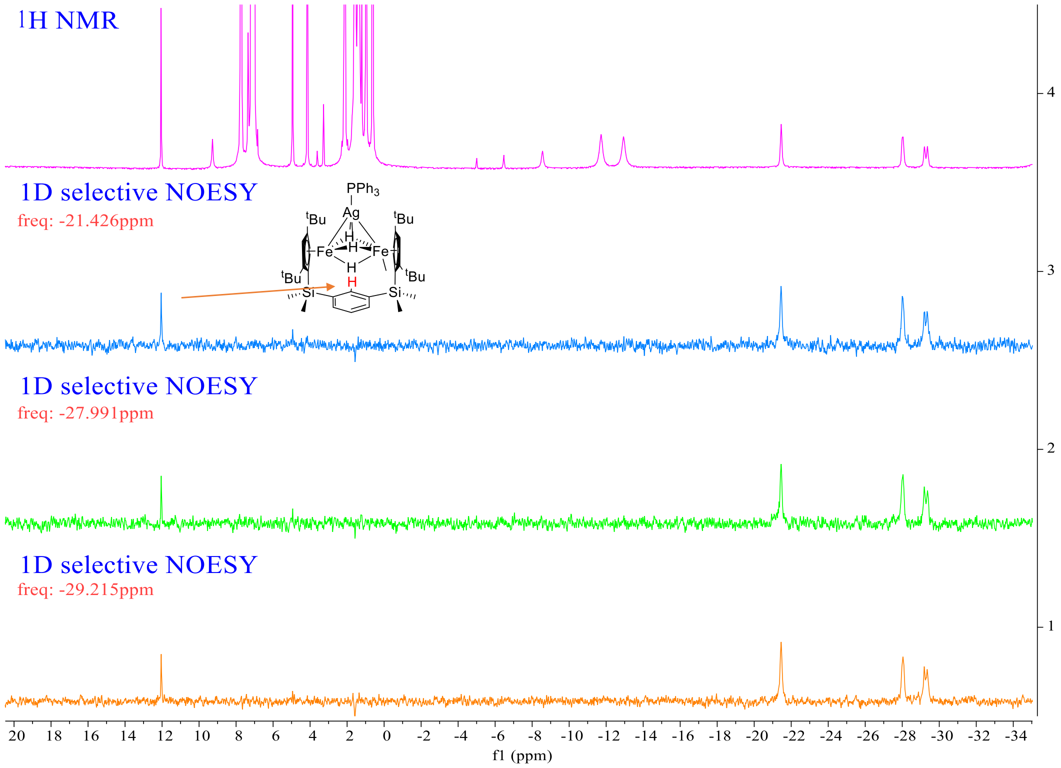

Figure S16. One-dimension selective ^1H NOSEY NMR spectrum of **LFe₂(μ-H)₃Ag(PPh₃) (5)** in toluene-d_8 at –40 °C.

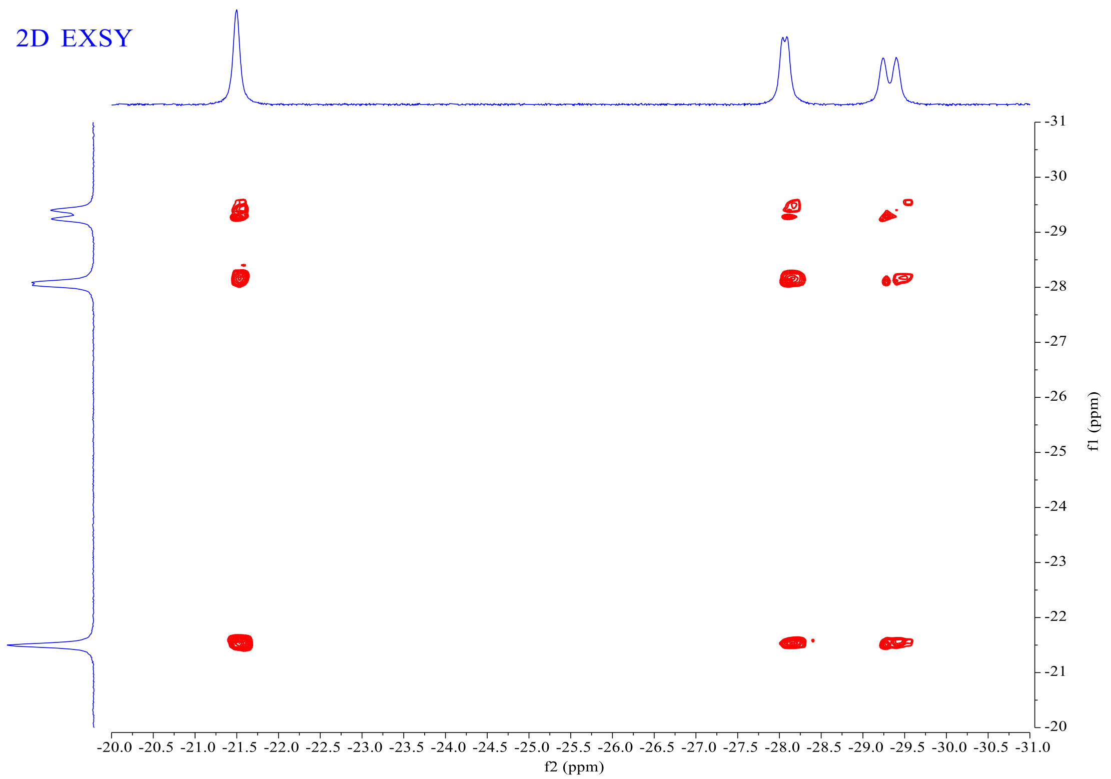

Figure S17. Partial ^{1}H (the hydride region) EXSY NMR spectrum of **$LFe_2(\mu\text{-}H)_3Ag(PPh_3)$ (5)** in toluene-d_8 at –40 °C.

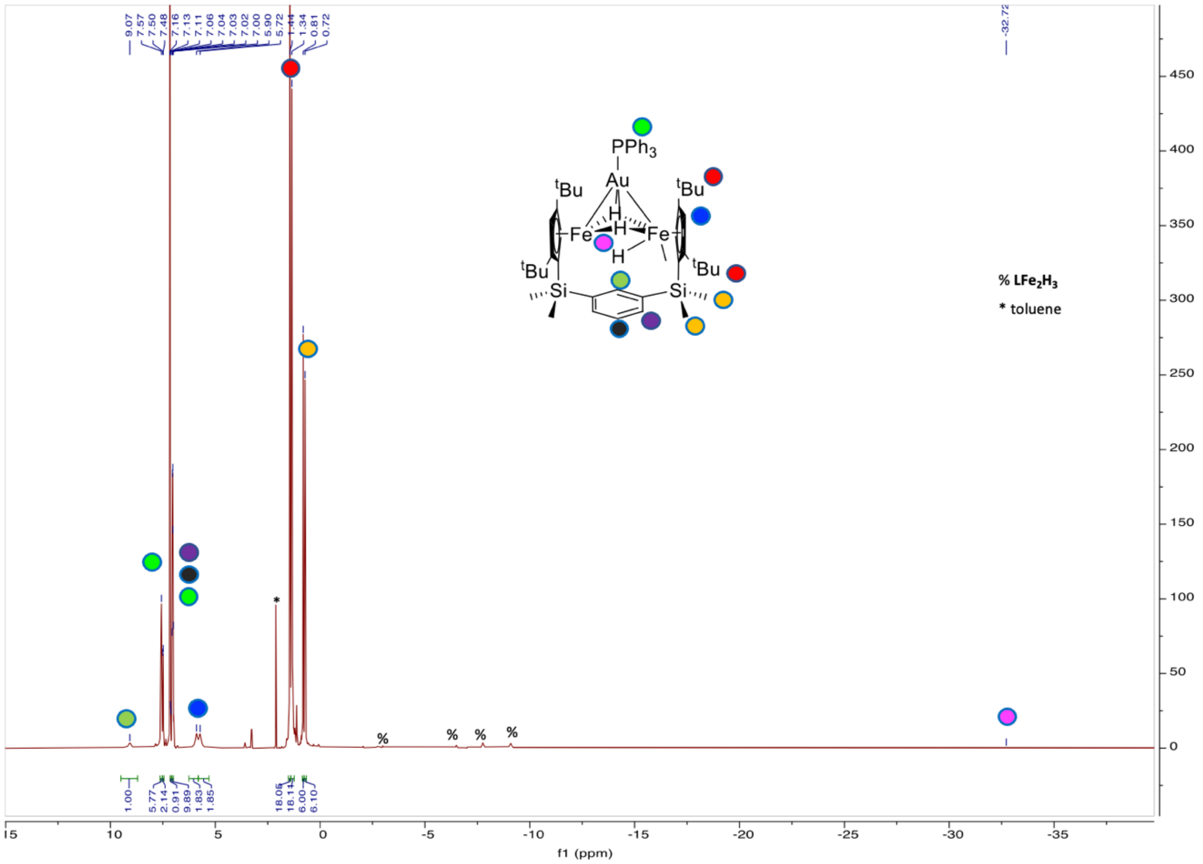

Figure S18. ^{1}H NMR spectrum of **$LFe_2(\mu\text{-}H)_3Au(PPh_3)$ (6)** in benzene-*d*$_6$.

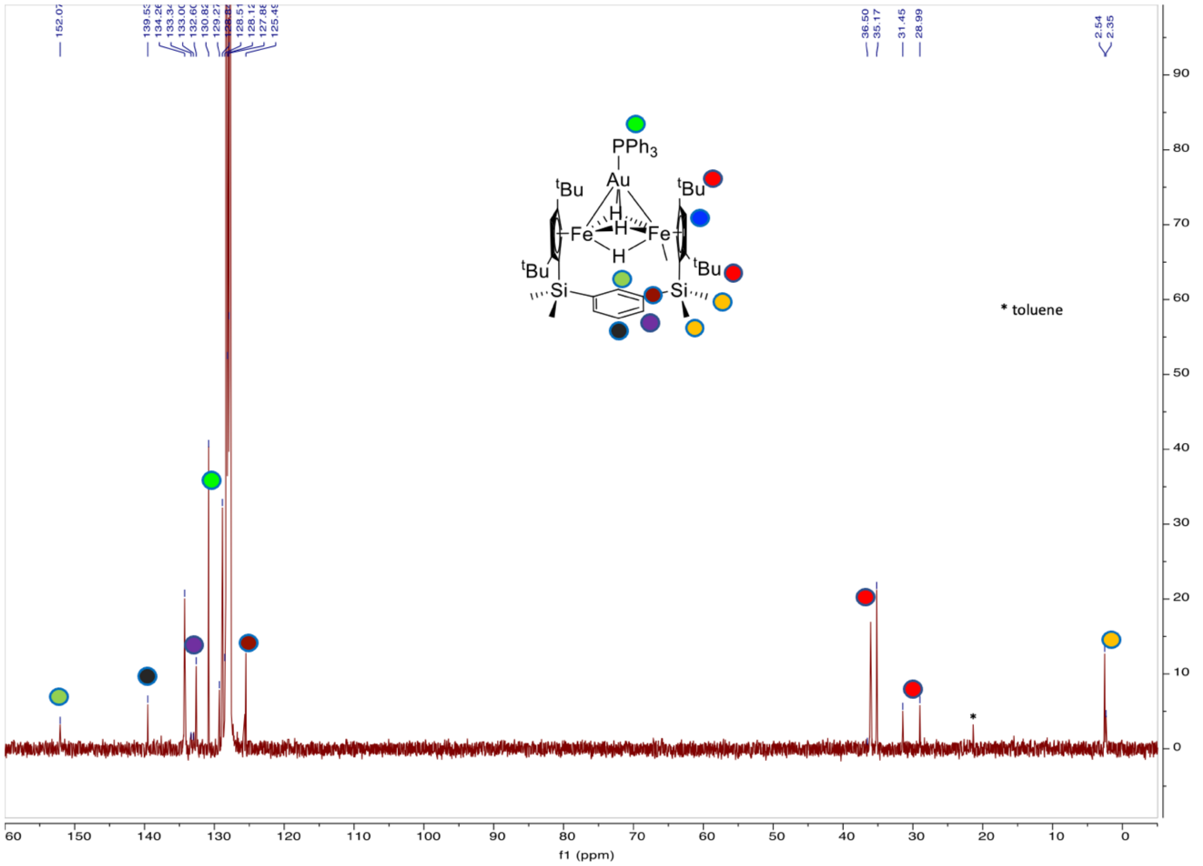

Figure S19. ^{13}C{^{1}H} NMR spectrum of **$LFe_2(\mu\text{-}H)_3Au(PPh_3)$ (6)** in benzene-*d*$_6$.

2. UV-vis spectra for all compounds

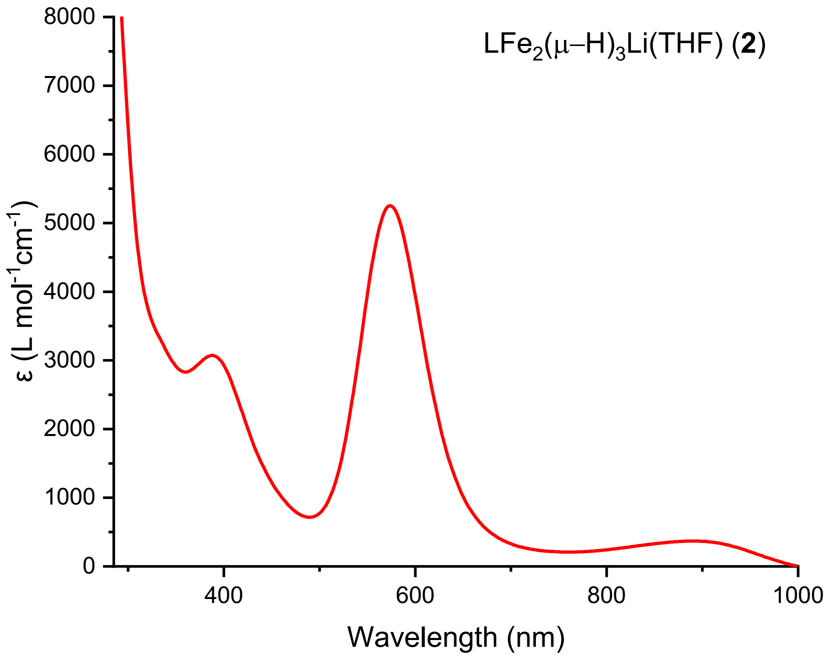

Figure S20. UV-Vis spectrum of **$LFe_2(\mu\text{-}H)_3Li(THF)$ (2)** in THF.

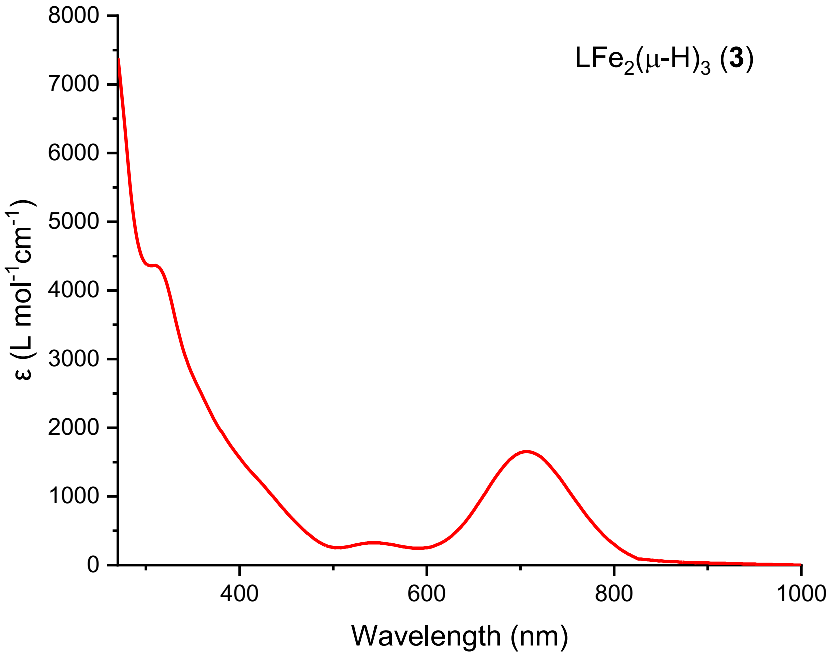

Figure S21. UV-Vis spectrum of **$LFe_2(\mu\text{-}H)_3$ (3)** in THF.

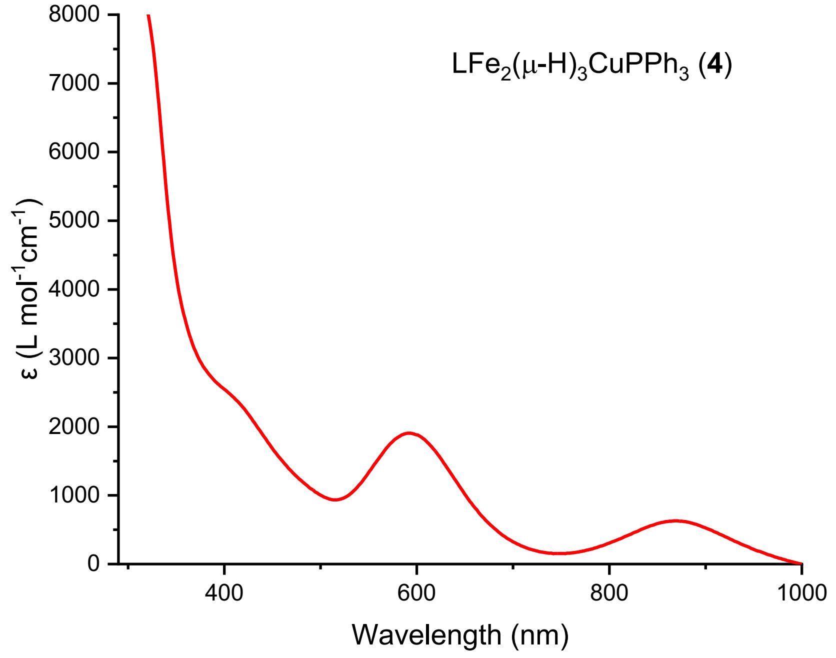

Figure S22. UV-Vis spectrum of **$LFe_2(\mu\text{-}H)_3Cu(PPh_3)$ (4)** in THF.

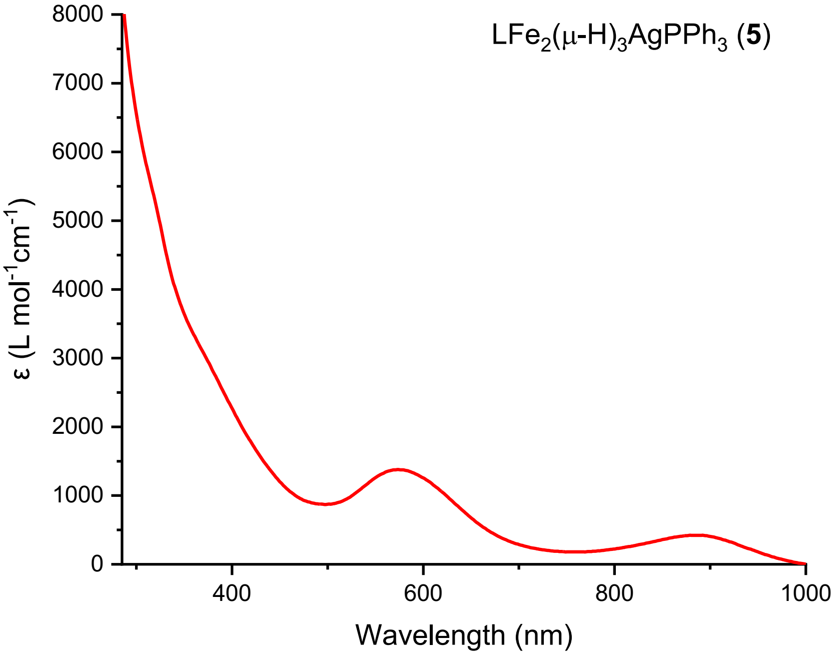

Figure S23. UV-Vis spectrum of **$LFe_2(\mu\text{-}H)_3Ag(PPh_3)$ (5)** in THF.

.

3. Single-crystal X-ray diffraction crystal structure figures and data tables

Single-crystal X-ray diffraction for complexes **1**, **3**-**6** were performed on a Bruker APEX DUO diffractometer with APEX II 4K and multi-layer mirror monochromated Mo K_α radiation (λ = 0.71073 Å) at 200(2) K. Data collection and reduction were performed with Bruker APEX II software. All of non-hydrogen atoms are refined anisotropically. Hydrogen atoms attached to the carbons were fixed at calculated positions and refined using a riding mode. Multiple disordered solvent molecules were observed in the crystal structures of all complexes. Whenever possible, co-crystallizing solvent molecules were modeled. Otherwise, SQUEEZE was employed to treat diffuse solvent contribution in the voids. For complex **2**, Single crystals data were collected on Rigaku XtaLABHyPix-Arc 150 diffractometer with Cu-Kα radiation (λ = 1.54178 Å) at 100(10) K. The structure determinations and refinements were carried out using the SHELXS and SHELXL programs, respectively, on the Olex2 interface. The structures were solved using direct methods, which yielded the positions of all non-hydrogen atoms. Hydrogen atoms on carbons were placed in calculated positions in the final structure refinement.

All cif files have been deposited on CCDC (2255895 (**1**), 2255896 (**2**), 2255898 (**3**), 2255892 (**4**), 2255893 (**5**), and 2255894 (**6**)).

Table S1. Comparison of selected bond lengths of complexes **2**-**6**.

	**2** (M = Li)	**3**	**4** (M = Cu)	**5** (M = Ag)	**6** (M = Au)
Fe···Fe contact (Å)	2.2391(5)	2.2210(5)	2.2659(9)	2.2513(14)	2.2725(10)
Avg. $Cp_{centroid}$–Fe	1.653	1.711	1.674	1.673	1.698
Avg. Fe···M contact (Å)	2.571(7)	-	2.5115(16)	2.6900(17)	2.6701(12)
M–H1 (Å) (shorter)	2.008	-	1.853	2.269	2.362
M–H2 (Å) (longer)	2.146	-	2.158	2.497	-[a]

***Table S1*.** Crystal data and structure refinement for **$LFe_2(\mu\text{-}Cl)_2$ (1)**

Empirical formula	$C_{36}H_{56}Cl_2Fe_2Si_2$	
Formula weight	727.59	
Temperature	200(2) K	
Wavelength	0.71073 Å	
Crystal system	Monoclinic	
Space group	P 21/n	
Unit cell dimensions	a = 9.0396(6) Å	a= 90°.
	b = 18.0528(12) Å	b= 96.929(3)°.
	c = 23.928(2) Å	g = 90°.
Volume	3876.4(5) $Å^3$	
Z	4	
Density (calculated)	1.247 Mg/m^3	
Absorption coefficient	0.970 mm^{-1}	
F(000)	1544	
Crystal size	0.57 x 0.36 x 0.10 mm^3	
Theta range for data collection	2.05 to 25.05°.	
Index ranges	-10<=h<=10, -21<=k<=21, -28<=l<=28	
Reflections collected	49072	
Independent reflections	6845 [R(int) = 0.0454]	
Completeness to theta = 25.05°	99.7 %	
Absorption correction	multi-scan	
Max. and min. transmission	0.9092 and 0.6077	
Refinement method	Full-matrix least-squares on F^2	
Data / restraints / parameters	6845 / 0 / 395	
Goodness-of-fit on F^2	1.004	
Final R indices [I>2sigma(I)]	R1 = 0.0330, wR2 = 0.0834	
R indices (all data)	R1 = 0.0422, wR2 = 0.0886	
Largest diff. peak and hole	0.689 and -0.300 e.$Å^{-3}$	

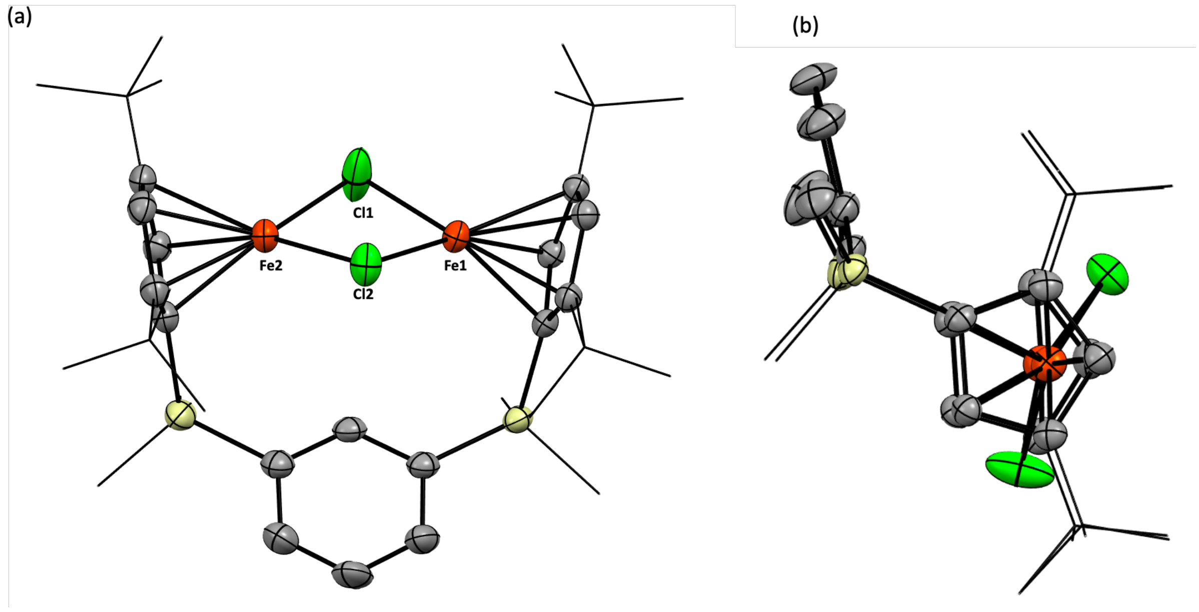

Figure S24. ORTEP of **LFe$_2$(μ-Cl)$_2$** (**1**). (a) The front view and (b) the side view of X-ray structure of complex **1**. 50% thermal ellipsoids; hydrogen atoms omitted for clarity). Selected interatomic distances (Å) and angles (º): Fe1–Fe2 3.234, Fe1–Cl1 2.3454(7), Fe1–Cl2 2.3382(7), Fe2–Cl1 2.3482(7), and Fe2–Cl2 2.3559(6); Fe1–Cl1–Fe2 87.11(2), and Fe1–Cl2–Fe2 87.10(2).

Table S2. Crystal data and structure refinement for **$LFe_2(\mu\text{-}H)_3LiTHF$ (2)**

Empirical formula	$C_{40}H_{67}Fe_2LiOSi_2$
Formula weight	738.75
Temperature/K	99.99(10)
Crystal system	monoclinic
Space group	P21/n
a/Å	13.64640(10)
b/Å	16.4690(2)
c/Å	23.2175(2)
α/°	90
β/°	95.0110(10)
γ/°	90
Volume/Å^3	5198.02(9)
Z	4
ρcalcg/cm^3	0.944
μ/mm 1	5.070
F(000)	1592.0
Crystal size/mm3	0.32 × 0.06 × 0.04
Radiation	Cu Kα (λ = 1.54184)
2Θ range for data collection/°	6.588 to 134.146
Index ranges	-15 ≤ h ≤ 16, -12 ≤ k ≤ 19, -26 ≤ l ≤ 27
Reflections collected	39410
Independent reflections	9249 [Rint = 0.0293, Rsigma = 0.0254]
Data/restraints/parameters	9249/0/431
Goodness-of-fit on F^2	1.099
Final R indexes [I>=2σ (I)]	R1 = 0.0442, wR2 = 0.1341
Final R indexes [all data]	R1 = 0.0487, wR2 = 0.1372
Largest diff. peak/hole / e Å^{-3}	0.42/-0.42

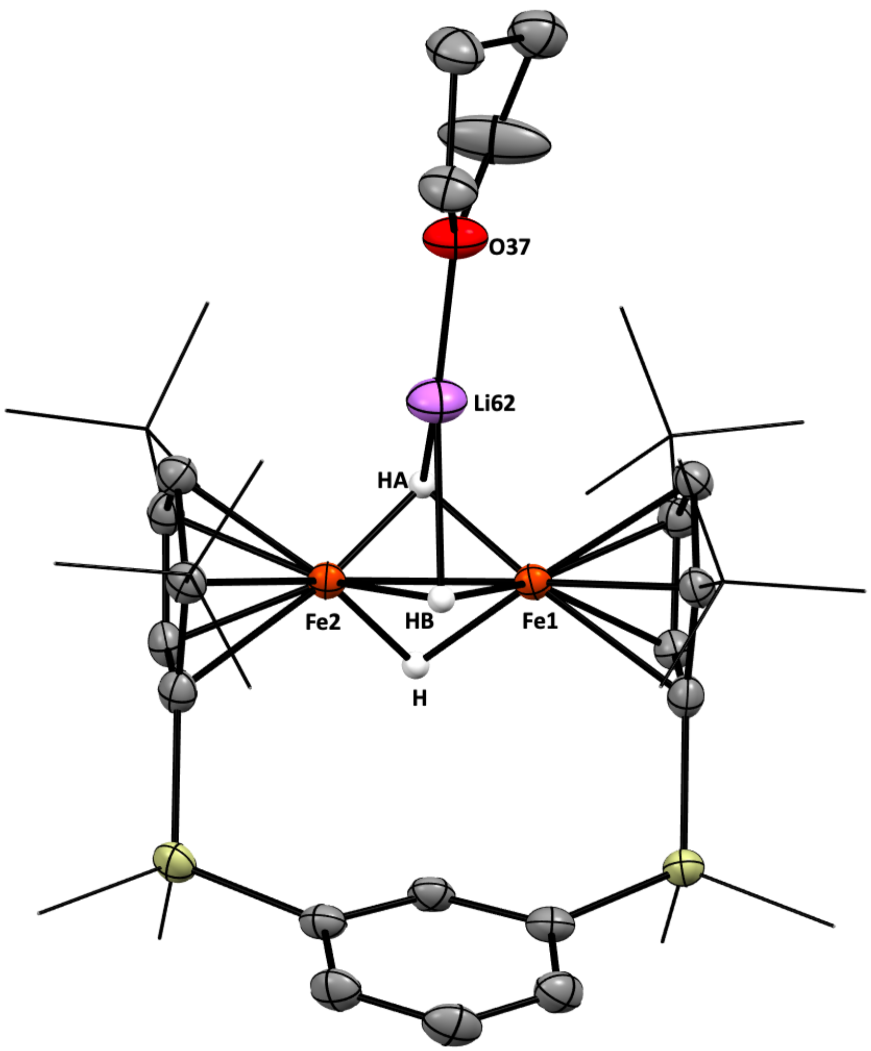

Figure S25. ORTEP of **LFe$_2$(μ-H)$_3$Li(THF)** (**2**)**.** (50% thermal ellipsoids; hydrogen atoms except metal hydrides (H, HA, and HB) omitted for clarity). Selected interatomic distances (Å): Fe1–Fe2 2.2391(5), Fe1–Li62 2.594(5), Fe2–Li62 2.548(5), Li62–O37 1.870(5), Fe1–H 1.724, Fe1–HA 1.653, Fe1–HB 1.648, Fe2–H 1.630, Fe2–HA 1.601, Fe2–HB 1.666, Li62–HA 2.008, and Li62–HB 2.146.

Table S3. Crystal data and structure refinement for **$LFe_2(\mu\text{-}H)_3$ (3)**

Empirical formula	$C_{36}H_{59}Fe_2Si_2$	
Formula weight	659.71	
Temperature	221(2) K	
Wavelength	0.71073 Å	
Crystal system	Monoclinic	
Space group	P 21/n	
Unit cell dimensions	a = 9.0711(10) Å	a= 90°.
	b = 18.933(2) Å	b= 100.886(4)°.
	c = 21.664(2) Å	g = 90°.
Volume	3653.7(7) Å^3	
Z	4	
Density (calculated)	1.199 Mg/m^3	
Absorption coefficient	0.881 mm^{-1}	
F(000)	1420	
Crystal size	0.14 x 0.14 x 0.06 mm^3	
Theta range for data collection	2.31 to 25.12°.	
Index ranges	-10<=h<=9, -22<=k<=22, -25<=l<=25	
Reflections collected	47423	
Independent reflections	6486 [R(int) = 0.0463]	
Completeness to theta = 25.12°	99.5 %	
Absorption correction	multi-scan	
Max. and min. transmission	0.9490 and 0.8865	
Refinement method	Full-matrix least-squares on F^2	
Data / restraints / parameters	6486 / 0 / 377	
Goodness-of-fit on F^2	1.032	
Final R indices [I>2sigma(I)]	R1 = 0.0302, wR2 = 0.0714	
R indices (all data)	R1 = 0.0422, wR2 = 0.0774	
Largest diff. peak and hole	0.306 and -0.367 e.Å^{-3}	

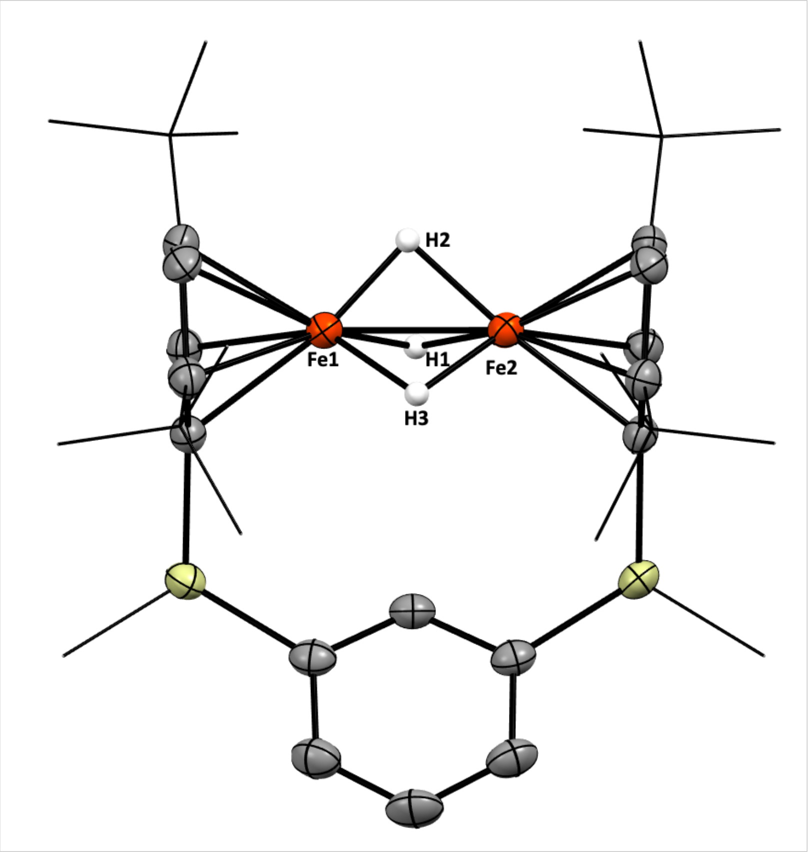

Figure S26. ORTEP of **$LFe_2(\mu\text{-}H)_3$** (**3**)**.** (50% thermal ellipsoids; hydrogen atoms except metal hydrides (H1, H2, and H3)omitted for clarity). Selected interatomic distances (Å): Fe1–Fe2 2.2210(5), Fe1–H1 1.6012, Fe1–H2 1.5247, Fe1–H3 1.6210, Fe2–H1 1.5863, Fe2–H2 1.6517, and Fe2–H3 1.5975.

Table S4. Crystal data and structure refinement for **$LFe_2(\mu\text{-}H)_3CuPPh_3$ (4)**

Empirical formula	$C_{54}H_{74}CuFe_2PSi_2$	
Formula weight	985.52	
Temperature	200(2) K	
Wavelength	0.71073 Å	
Crystal system	Triclinic	
Space group	P -1	
Unit cell dimensions	a = 12.591(5) Å	a= 91.522(17)°.
	b = 14.409(7) Å	b= 110.319(14)°.
	c = 17.136(6) Å	g = 115.863(14)°.
Volume	2564.4(19) $Å^3$	
Z	2	
Density (calculated)	1.276 Mg/m^3	
Absorption coefficient	1.081 mm^{-1}	
F(000)	1044	
Crystal size	0.75 x 0.70 x 0.68 mm^3	
Theta range for data collection	2.75 to 25.04°.	
Index ranges	-14<=h<=14, -17<=k<=17, -20<=l<=20	
Reflections collected	77712	
Independent reflections	9006 [R(int) = 0.0383]	
Completeness to theta = 25.04°	99.2 %	
Absorption correction	multi-scan	
Max. and min. transmission	0.5269 and 0.4978	
Refinement method	Full-matrix least-squares on F^2	
Data / restraints / parameters	9006 / 0 / 557	
Goodness-of-fit on F2	1.023	
Final R indices [I>2sigma(I)]	R1 = 0.0258, wR2 = 0.0659	
R indices (all data)	R1 = 0.0286, wR2 = 0.0678	
Largest diff. peak and hole	0.447 and -0.287 e.$Å^{-3}$	

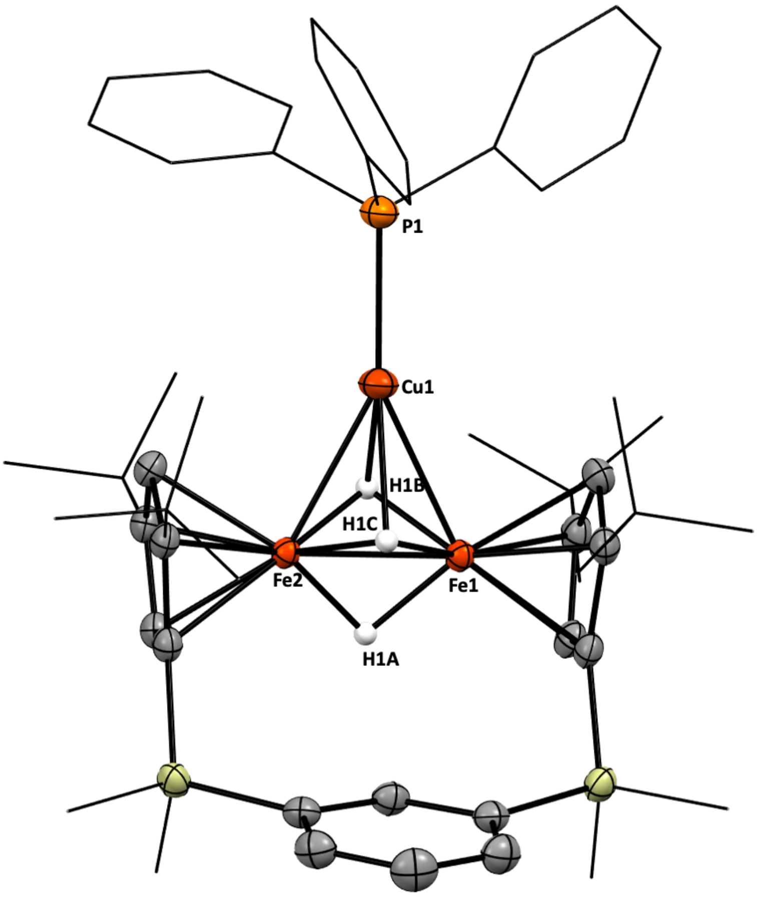

Figure S27. ORTEP of **$LFe_2(\mu\text{-}H)_3Cu(PPh_3)$** (**4**). (50% thermal ellipsoids; hydrogen atoms except metal hydrides (H1A, H1B, and H1C) omitted for clarity). Selected interatomic distances (Å): Fe1–Fe2 2.2659(9), Fe1–Cu1 2.5213(11), Fe2–Cu1 2.5017(12), Cu1–P1 2.2308(10), Fe1–H1A 1.6076, Fe1–H1B 1.6294, Fe1–H1C 1.6549, Fe2–H1A 1.5855, Fe2–H1B 1.6606, Fe2–H1C 1.6757, Cu1–H1B 1.8530, and Cu1–H1C 2.158.

Table S5. Crystal data and structure refinement for **$LFe_2(\mu\text{-}H)_3AgPPh_3$ (5)**

Empirical formula	$C_{54}H_{74}AgFe_2PSi_2$	
Formula weight	1029.85	
Temperature	200(2) K	
Wavelength	0.71073 Å	
Crystal system	Triclinic	
Space group	P -1	
Unit cell dimensions	a = 12.517(2) Å	a= 72.734(3)°.
	b = 14.455(2) Å	b= 70.394(4)°.
	c = 17.142(2) Å	g = 64.541(4)°.
Volume	2595.3(7) $Å^3$	
Z	2	
Density (calculated)	1.318 Mg/m^3	
Absorption coefficient	1.035 mm^{-1}	
F(000)	1080	
Crystal size	0.58 x 0.06 x 0.01 mm^3	
Theta range for data collection	2.21 to 25.01°.	
Index ranges	-14<=h<=14, -17<=k<=17, -20<=l<=20	
Reflections collected	54389	
Independent reflections	9116 [R(int) = 0.1528]	
Completeness to theta = 25.01°	99.7 %	
Absorption correction	multi-scan	
Max. and min. transmission	0.9897 and 0.5850	
Refinement method	Full-matrix least-squares on F^2	
Data / restraints / parameters	9116 / 0 / 557	
Goodness-of-fit on F2	1.011	
Final R indices [I>2sigma(I)]	R1 = 0.0719, wR2 = 0.1540	
R indices (all data)	R1 = 0.1400, wR2 = 0.1870	
Largest diff. peak and hole	0.787 and -1.175 e.$Å^{-3}$	

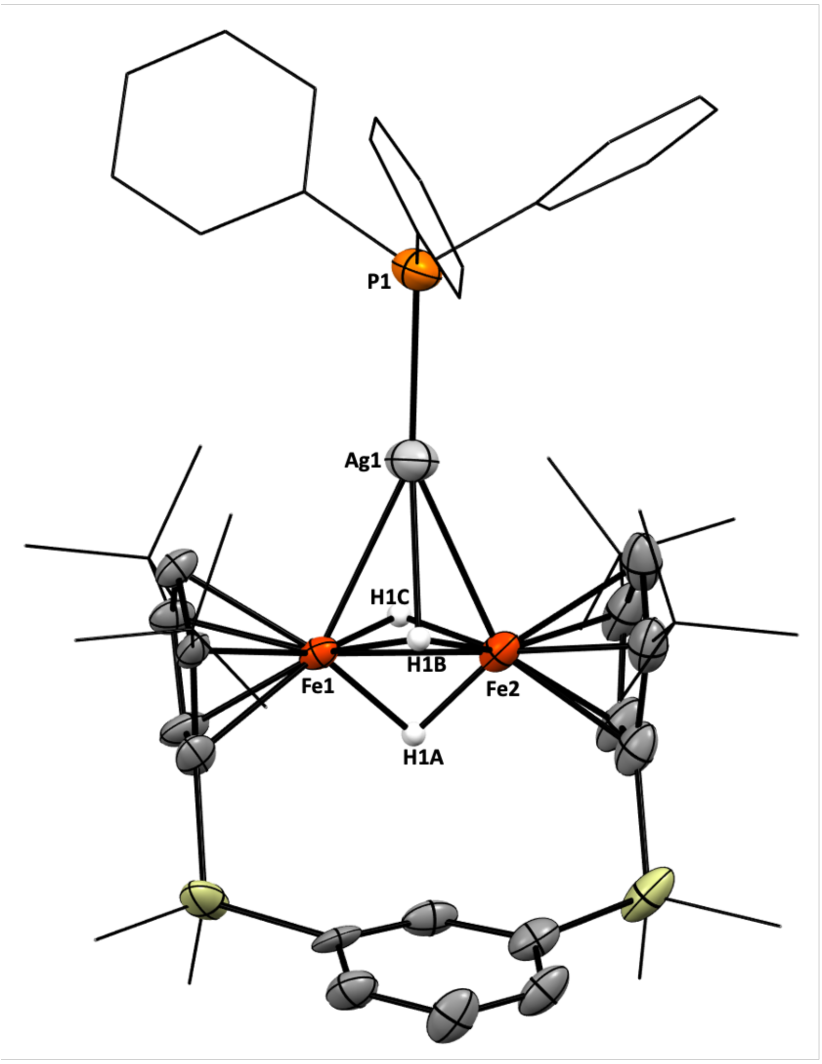

Figure S28. ORTEP of **$LFe_2(\mu\text{-}H)_3Ag(PPh_3)$ (5).** (50% thermal ellipsoids; hydrogen atoms except metal hydrides (H1A, H1B, and H1C) omitted for clarity). Selected interatomic distances (Å): Fe1–Fe2 2.2513(14), Fe1–Ag1 2.6817(12), Fe2–Ag1 2.6982(12), Ag1–P1 2.423(2), Fe1–H1A 1.7268, Fe1–H1B 1.6165, Fe1–H1C 1.5720, Fe2–H1A 1.5656, Fe2–H1B 1.6481, Fe2–H1C 1.5821, Ag1–H1B 2.269, and Ag1–H1C 2.497.

Table S6. Crystal data and structure refinement for **$LFe_2(\mu\text{-}H)_3AuPPh_3$ (6)**

Empirical formula	$C_{54}H_{73}AuFe_2PSi_2$	
Formula weight	1117.94	
Temperature	200(2) K	
Wavelength	0.71073 Å	
Crystal system	Triclinic	
Space group	P -1	
Unit cell dimensions	a = 12.562(2) Å	a= 72.620(5)°.
	b = 14.393(3) Å	b= 70.487(5)°.
	c = 17.157(3) Å	g = 64.850(4)°.
Volume	2601.5(8) Å^3	
Z	2	
Density (calculated)	1.427 Mg/m^3	
Absorption coefficient	3.473 mm^{-1}	
F(000)	1142	
Crystal size	0.29 x 0.11 x 0.02 mm^3	
Theta range for data collection	2.22 to 25.29°.	
Index ranges	-15<=h<=14, -17<=k<=17, -20<=l<=20	
Reflections collected	53486	
Independent reflections	9365 [R(int) = 0.0737]	
Completeness to theta = 25.29°	98.8 %	
Absorption correction	multi-scan	
Max. and min. transmission	0.9338 and 0.4324	
Refinement method	Full-matrix least-squares on F^2	
Data / restraints / parameters	9365 / 0 / 537	
Goodness-of-fit on F2	1.025	
Final R indices [I>2sigma(I)]	R1 = 0.0400, wR2 = 0.0853	
R indices (all data)	R1 = 0.0579, wR2 = 0.0923	
Largest diff. peak and hole	0.826 and -0.896 e.Å^{-3}	

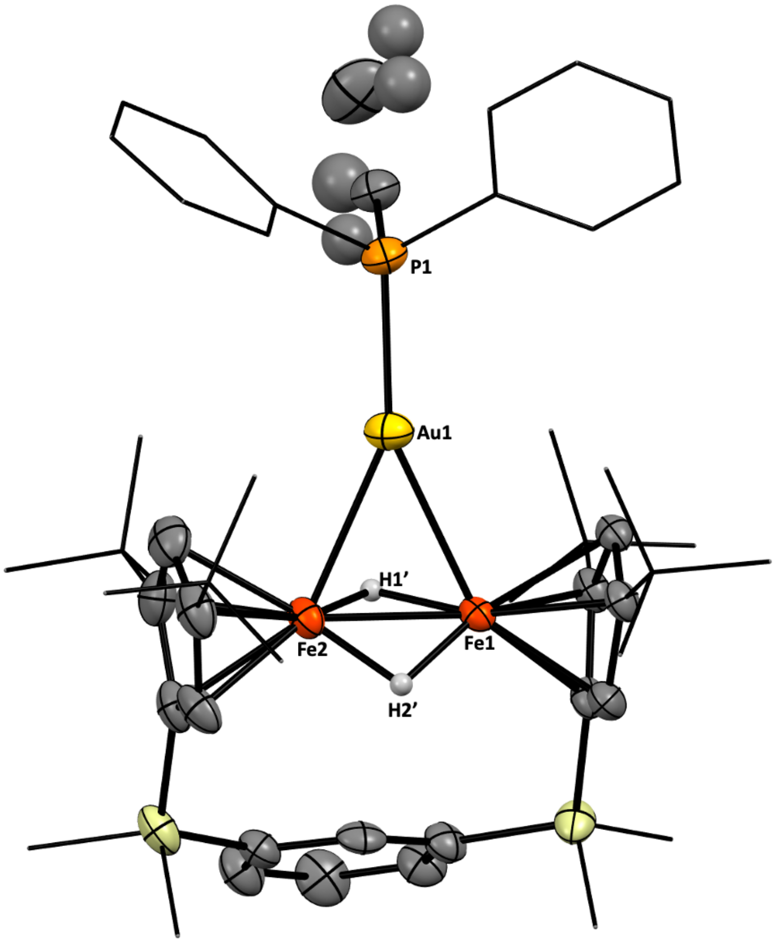

Figure S29. ORTEP of **LFe$_2$(μ-H)$_3$Au(PPh$_3$)** (**6**)**.** (50% thermal ellipsoids; hydrogen atoms except metal hydrides (H1A, H1B, and H1C) omitted for clarity). Selected interatomic distances (Å): Fe1–Fe2 2.2725(10), Fe1–Au1 2.6645(9), Fe2–Au1 2.6756(8), Au1–P1 2.2900(15), Fe1–H1' 1.6458, Fe1–H2' 1.5492, Fe2–H1' 1.5819, Fe2–H2' 1.5494, Au1–H1' 2.362, and Au1–H2' 3.350.

4. IR spectra

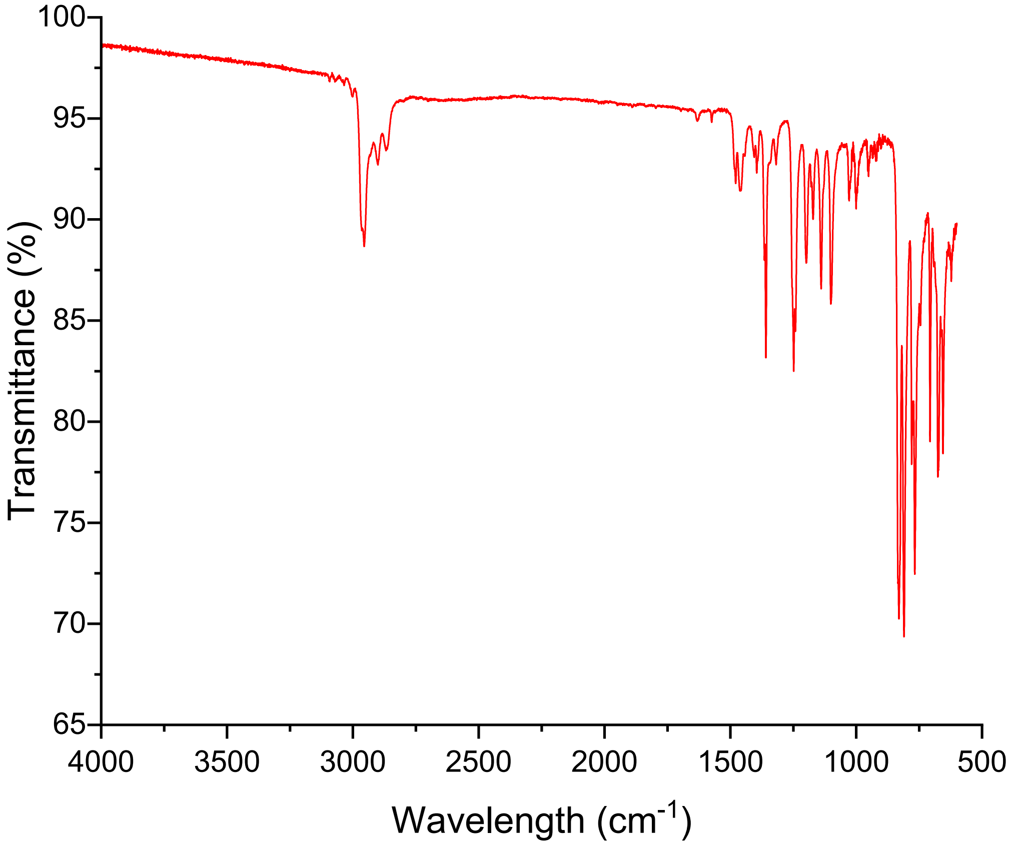

Figure S30. IR spectrum of **LFe_2Cl_2 (1)**.

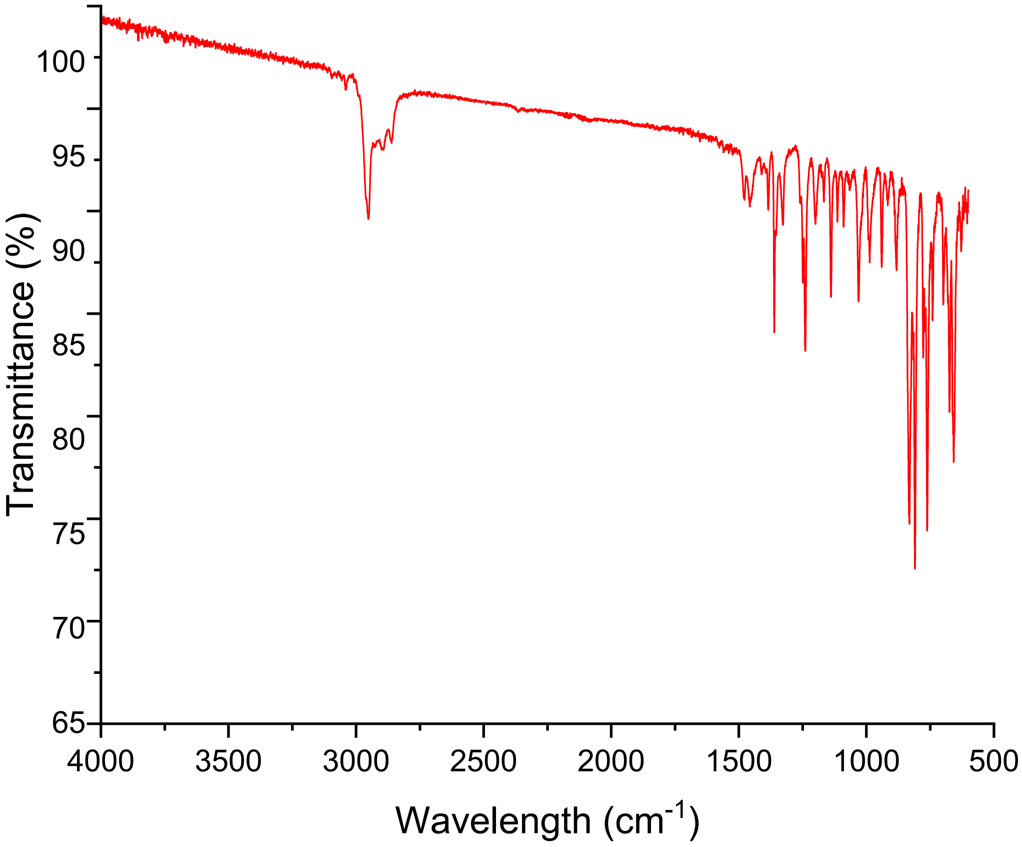

Figure S31. IR spectrum of **$LFe_2(\mu\text{-}H)_3Li(THF)$** (**2**).

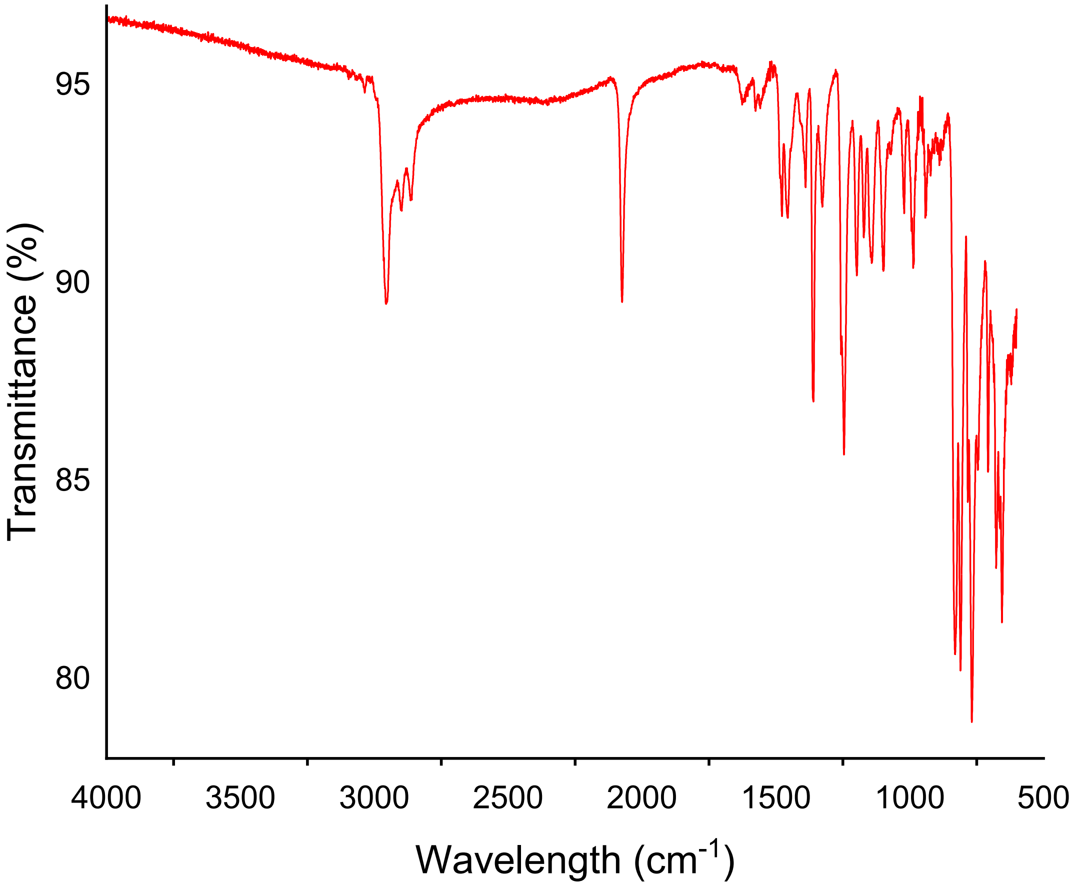

Figure S32. IR spectrum of **$LFe_2(\mu$-$H)_3$** (**3**).

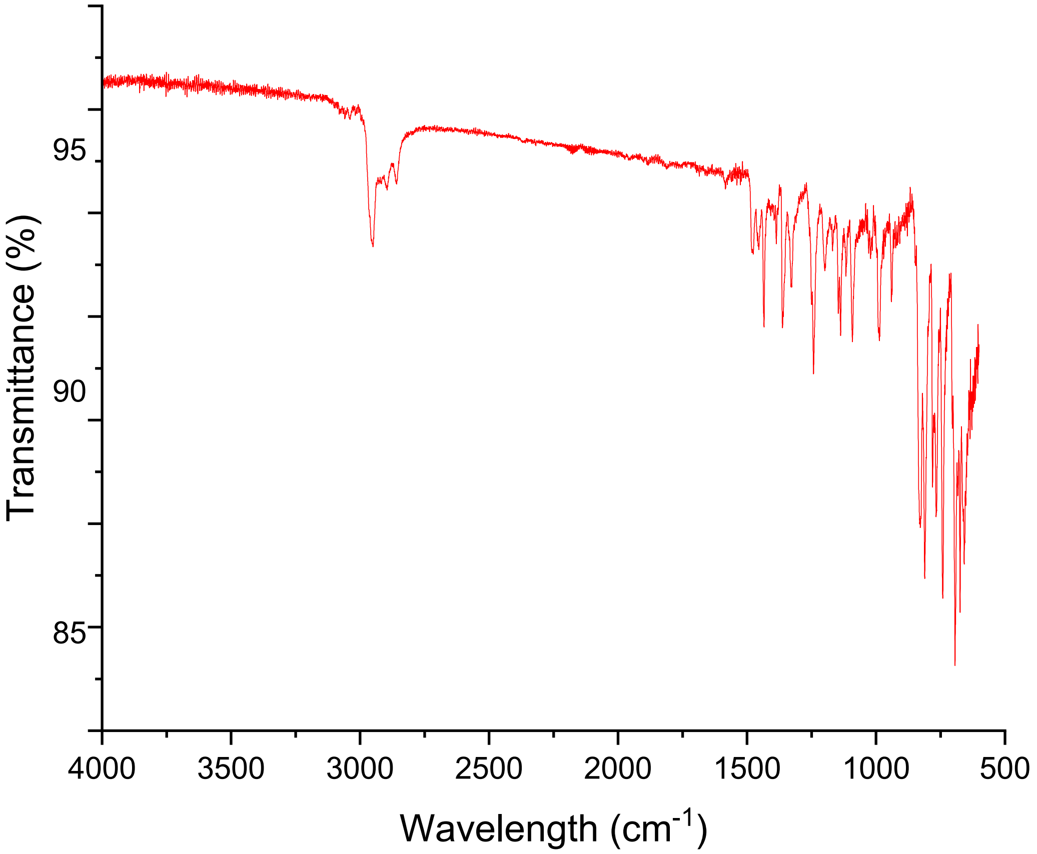

Figure S33. IR spectrum of **$LFe_2(\mu$-$H)_3Cu(PPh_3)$ (4)**.

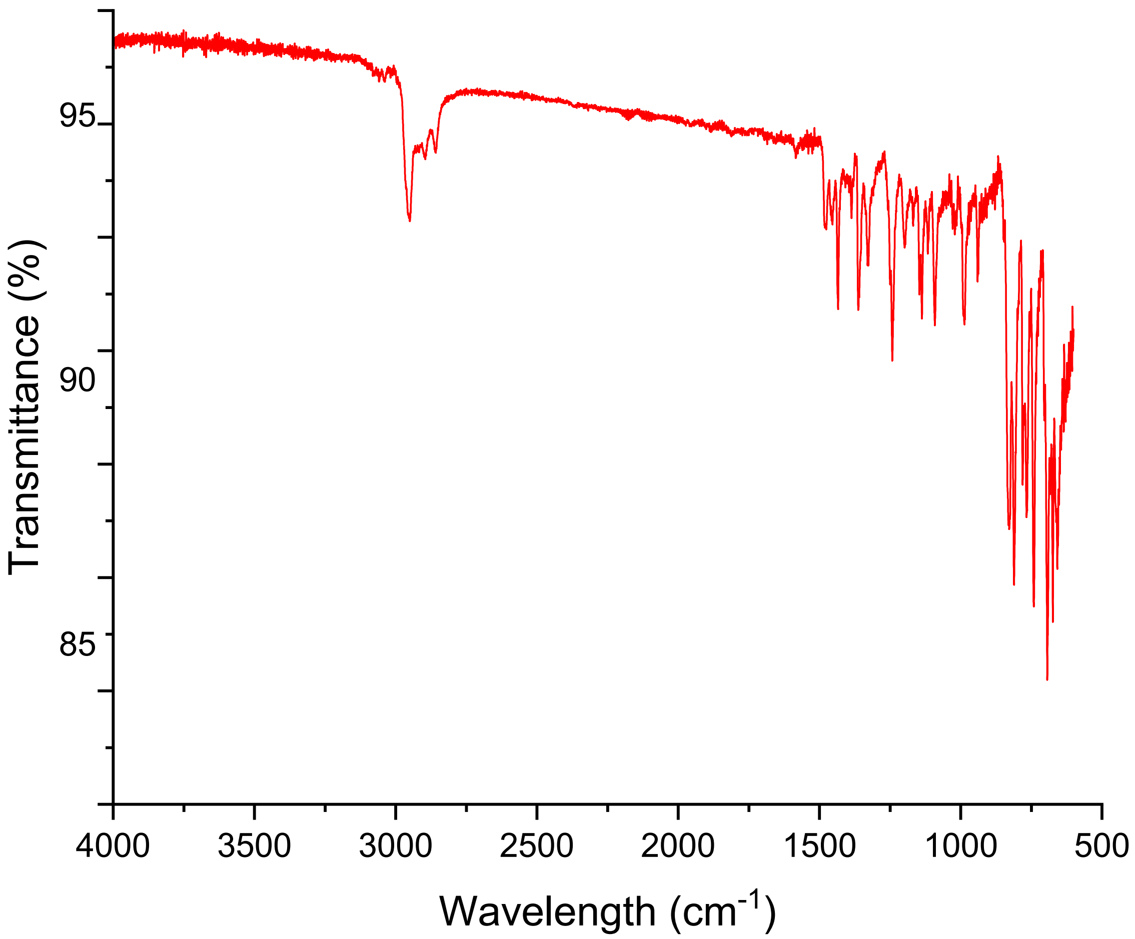

Figure S34. IR spectrum of **$LFe_2(\mu\text{-}H)_3Ag(PPh_3)$ (5)**.

5. Computational results

Density Functional Theory (DFT) was applied by the means of the B3LYP hybrid functional,[1-4] corrected for dispersion as proposed by Grimme (D3 correction, BJ damping).[5-6] Calculations were performed with the Gaussian 16 suite of programs.[7] Geometry optimizations were performed without any symmetry constraints using the effective core potential basis set LanL2DZ[8-10] on Li, Fe, Ag and 6-31G(d,p)[11-12] on the rest of the atoms. NBO analyses were performed with NBO 6.0.[13]

6. References

1. Becke, A. D., Density‐functional thermochemistry. III. The role of exact exchange. *J. Chem. Phys.* **1993,** *98* (7), 5648-5652.
2. Lee, C.; Yang, W.; Parr, R. G., Development of the Colle-Salvetti correlation-energy formula into a functional of the electron density. *Physical Review B* **1988,** *37* (2), 785-789.
3. Vosko, S. H.; Wilk, L.; Nusair, M., Accurate spin-dependent electron liquid correlation energies for local spin density calculations: a critical analysis. *Canadian Journal of Physics* **1980,** *58* (8), 1200-1211.
4. Stephens, P. J.; Devlin, F. J.; Chabalowski, C. F.; Frisch, M. J., Ab Initio Calculation of Vibrational Absorption and Circular Dichroism Spectra Using Density Functional Force Fields. *The Journal of Physical Chemistry* **1994,** *98* (45), 11623-11627.
5. Grimme, S.; Antony, J.; Ehrlich, S.; Krieg, H., A consistent and accurate ab initio parametrization of density functional dispersion correction (DFT-D) for the 94 elements H-Pu. *J. Chem. Phys.* **2010,** *132* (15), 154104.
6. Grimme, S.; Ehrlich, S.; Goerigk, L., Effect of the damping function in dispersion corrected density functional theory. *J. Comput. Chem.* **2011,** *32* (7), 1456-1465.
7. Frisch, M. J.; Trucks, G. W.; Schlegel, H. B.; Scuseria, G. E.; Robb, M. A.; Cheeseman, J. R.; Scalmani, G.; Barone, V.; Petersson, G. A.; Nakatsuji, H.; Li, X.; Caricato, M.; Marenich, A. V.; Bloino, J.; Janesko, B. G.; Gomperts, R.; Mennucci, B.; Hratchian, H. P.; Ortiz, J. V.; Izmaylov, A. F.; Sonnenberg, J. L.; Williams; Ding, F.; Lipparini, F.; Egidi, F.; Goings, J.; Peng, B.; Petrone, A.; Henderson, T.; Ranasinghe, D.; Zakrzewski, V. G.; Gao, J.; Rega, N.; Zheng, G.; Liang, W.; Hada, M.; Ehara, M.; Toyota, K.; Fukuda, R.; Hasegawa, J.; Ishida, M.; Nakajima, T.; Honda, Y.; Kitao, O.; Nakai, H.; Vreven, T.; Throssell, K.; Montgomery Jr., J. A.; Peralta, J. E.; Ogliaro, F.; Bearpark, M. J.; Heyd, J. J.; Brothers, E. N.; Kudin, K. N.; Staroverov, V. N.; Keith, T. A.; Kobayashi, R.; Normand, J.; Raghavachari, K.; Rendell, A. P.; Burant, J. C.; Iyengar, S. S.; Tomasi, J.; Cossi, M.; Millam, J. M.; Klene, M.; Adamo, C.; Cammi, R.; Ochterski, J. W.; Martin, R. L.; Morokuma, K.; Farkas, O.; Foresman, J. B.; Fox, D. J. *Gaussian 16 Rev. C.01*, Wallingford, CT, 2016.
8. Hay, P. J.; Wadt, W. R., Ab initio effective core potentials for molecular calculations. Potentials for the transition metal atoms Sc to Hg. *J. Chem. Phys.* **1985,** *82* (1), 270-283.
9. Wadt, W. R.; Hay, P. J., Ab initio effective core potentials for molecular calculations. Potentials for main group elements Na to Bi. *J. Chem. Phys.* **1985,** *82* (1), 284-298.
10. Hay, P. J.; Wadt, W. R., Ab initio effective core potentials for molecular calculations. Potentials for K to Au including the outermost core orbitals. *J. Chem. Phys.* **1985,** *82* (1), 299-310.
11. Feller, D., The role of databases in support of computational chemistry calculations. *J. Comput. Chem.* **1996,** *17* (13), 1571-1586.
12. Schuchardt, K. L.; Didier, B. T.; Elsethagen, T.; Sun, L.; Gurumoorthi, V.; Chase, J.; Li, J.; Windus, T. L., Basis Set Exchange: A Community Database for Computational Sciences. *J. Chem. Inf. Model.* **2007,** *47* (3), 1045-1052.
13. Glendening, E. D.; Landis, C. R.; Weinhold, F., NBO 6.0: Natural bond orbital analysis program. *J. Comput. Chem.* **2013,** *34* (16), 1429-1437.
